# Crystallographic fragment-binding studies of the *Mycobacterium tuberculosis* trifunctional enzyme suggest binding pockets for the tails of the acyl-CoA substrates at its active sites and a potential substrate-channeling path between them

**DOI:** 10.1107/S2059798324006557

**Published:** 2024-07-16

**Authors:** Subhadra Dalwani, Alexander Metz, Franziska U. Huschmann, Manfred S. Weiss, Rik K. Wierenga, Rajaram Venkatesan

**Affiliations:** a Faculty of Biochemistry and Molecular Medicine University of Oulu Oulu Finland; b Department of Pharmaceutical Chemistry Philipps-University Marburg Marburg Germany; c Macromolecular Crystallography Helmholtz-Zentrum Berlin Berlin Germany; F. Hoffmann-La Roche Ltd, Switzerland

**Keywords:** CoA-dependent enzymes, electrostatic surfaces, lipid metabolism, substrate channeling, tuberculosis, fatty-acid β-oxidation, *Mycobacterium tuberculosis*, trifunctional enzyme

## Abstract

The *Mycobacterium tuberculosis* trifunctional enzyme (MtTFE) is an α_2_β_2_ tetrameric enzyme in which the α-chain harbors the 2*E*-enoyl-CoA hydratase (ECH) and 3*S*-hydroxyacyl-CoA dehydrogenase (HAD) active sites, and the β-chain provides the 3-ketoacyl-CoA thiolase (KAT) active site. Linear, medium-chain and long-chain 2*E*-enoyl-CoA molecules are the preferred substrates of MtTFE. Previous crystallographic binding and modeling studies identified binding sites for the acyl-CoA substrates at the three active sites, as well as the NAD binding pocket at the HAD active site. These studies also identified three additional CoA binding sites on the surface of MtTFE that are different from the active sites. It has been proposed that one of these additional sites could be of functional relevance for the substrate channeling (by surface crawling) of reaction intermediates between the three active sites. Here, 226 fragments were screened in a crystallographic fragment-binding study of MtTFE crystals, resulting in the structures of 16 MtTFE–fragment complexes. Analysis of the 121 fragment-binding events shows that the ECH active site is the ‘binding hotspot’ for the tested fragments, with 41 binding events. The mode of binding of the fragments bound at the active sites provides additional insight into how the long-chain acyl moiety of the substrates can be accommodated at their proposed binding pockets. In addition, the 20 fragment-binding events between the active sites identify potential transient binding sites of reaction intermediates relevant to the possible channeling of substrates between these active sites. These results provide a basis for further studies to understand the functional relevance of the latter binding sites and to identify substrates for which channeling is crucial.

## Abstract 2

Crystallographic fragment-binding studies of the *Mycobacterium tuberculosis* trifunctional enzyme have resulted in 121 binding events of 16 out of 226 investigated fragments, suggesting functional sites with respect to substrate binding and substrate channeling.

## 1. Introduction

**Figure 1:**
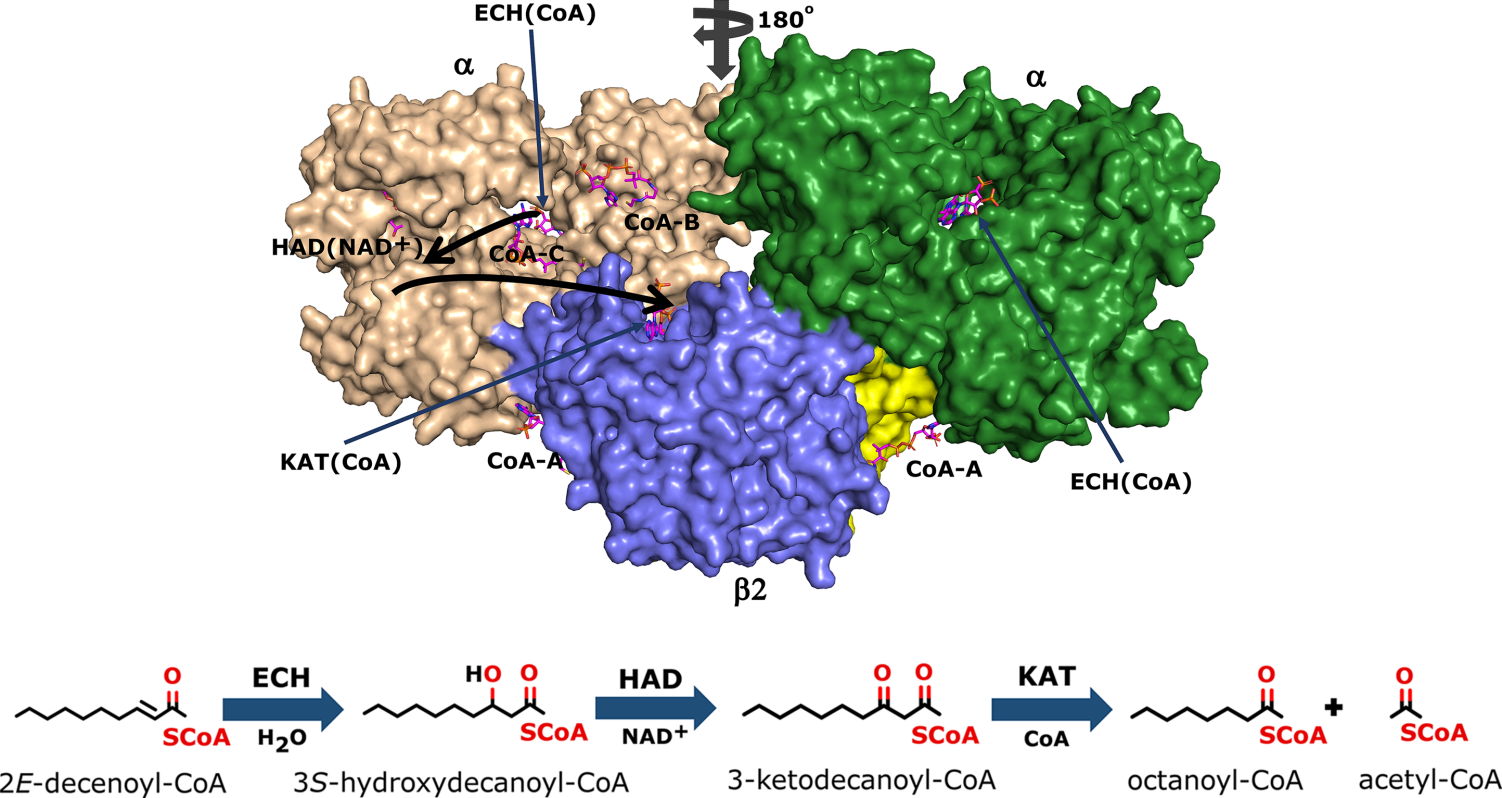
The TFE tetramer with its ECH, HAD and KAT active sites. Top: the two α-chains (wheat and green) are assembled on top of the β_2_ thiolase dimer (cyan, yellow). The vertical arrow visualizes the twofold axis of the α_2_β_2_ tetramer. The ECH, HAD and KAT active sites are labeled ECH(CoA) (with a thin arrow), HAD(NAD^+^) and KAT(CoA). CoAs bound at the additional CoA binding sites are labeled CoA-A, CoA-B and CoA-C. The curved thick arrows identify the path between the ECH and HAD active sites and between the HAD and KAT active sites. Bottom: schematic representation of the reactions catalyzed by the ECH, HAD and KAT active sites.

A large portion of the genome of *Mycobacterium tuberculosis* (*Mtb*) codes for enzymes involved in lipid metabolism (Cole *et al.*, 1998 ▸). Our understanding of the importance of lipid metabolism at the various stages of infection by *Mtb* has improved over the years, and several enzymes involved in the pathways of lipid metabolism are possible drug targets (Mi *et al.*, 2022 ▸). It has been shown that the genes that code for fatty-acid metabolism in general, as well as specifically for the β-oxidation pathway, are upregulated during the intracellular stages of infection (Rohde *et al.*, 2012 ▸; Schnappinger *et al.*, 2003 ▸). *Mtb* is capable of switching its metabolic preference during the latent stage of infection in order to utilize host-derived fatty acids as a source of carbon rather than glucose or glycerol (Wilburn *et al.*, 2018 ▸). The *Mtb* genome encodes an unusually large set of enzymes of the β-oxidation cycle (Cole *et al.*, 1998 ▸); however, only a single trifunctional enzyme (MtTFE) that catalyzes three of the last four reactions in the β-oxidation cycle (Fig. 1 ▸) is present in *Mtb*, in contrast to other bacteria such as *Escherichia coli*, which has two TFEs (Sah-Teli *et al.*, 2019 ▸, 2023 ▸). MtTFE has been characterized as an α_2_β_2_ heterotetrameric multifunctional enzyme complex (Fig. 1 ▸) encoded by the *fadA* (β subunit) and *fadB* (α subunit) genes (Venkatesan & Wierenga, 2013 ▸).

Bioinformatics studies have suggested that four TFE sub­families exist: (i) mammalian mitochondrial TFEs, (ii) the mycobacterial TFE (MtTFE), (iii) the bacterial aerobic TFEs from *E. coli* (EcTFE) and *Pseudomonas fragi* (PfTFE), and (iv) the bacterial anaerobic TFE (from *E. coli*; anEcTFE) (Venkatesan & Wierenga, 2013 ▸). Experimental evidence for the presence of substrate channeling by the multifunctional enzymes of the β-oxidation pathway for specific substrates exists for the rat peroxisomal multifunctional enzyme (type 1) (RnMFE1; Yang *et al.*, 1986 ▸), EcTFE (Yang *et al.*, 1986 ▸), mitochondrial TFE from pig heart (Yao & Schulz, 1996 ▸) and mitochondrial TFE from human (HsTFE; Nada *et al.*, 1995 ▸). While biochemical assays provide kinetic evidence for the existence of substrate channeling in a given multifunctional enzyme system, structural studies are essential to provide insight into a possible mechanism and to identify the transient binding sites. Substrate channeling offers several advantages in cellular metabolism, for example in cases where the intermediates are toxic or labile, or for facilitating the progress of reactions despite a highly unfavorable equilibrium (Wheeldon *et al.*, 2016 ▸; Sweetlove & Fernie, 2018 ▸). The mechanism of substrate channeling is dependent on the nature of the substrate. For small molecules it involves substrate tunneling through the matrix of the protein, for example in the well studied bifunctional enzyme tryptophan synthase (Barends *et al.*, 2008 ▸; Bosken *et al.*, 2022 ▸; D’Amico & Boehr, 2023 ▸), and for large polar molecules it involves surface crawling (over bulk solvent-exposed surface area), as observed for the bifunctional enzyme thymidylate synthase–dihydrofolate reductase (TS-DHFR; Anderson, 2017 ▸). In some cases, partial channeling of substrates has been observed (Baker *et al.*, 2012 ▸), in which case not all intermediates reach the subsequent active site by surface crawling, but a certain fraction diffuses via bulk solvent to the subsequent active site.

Each of the β-oxidation TFEs forms α_2_β_2_ heterotetramers (molecular mass 240 kDa) in which the β_2_ dimer forms the core of the tetramer; however, their quaternary assemblies differ due to different modes of assembly of the α chains onto the β_2_ dimer (Ishikawa *et al.*, 2004 ▸; Liang *et al.*, 2018 ▸; Sah-Teli *et al.*, 2020 ▸, 2023 ▸; Venkatesan & Wierenga, 2013 ▸; Xia *et al.*, 2019 ▸). MtTFE, EcTFE and PfTFE are soluble enzymes, whereas HsTFE and anEcTFE are membrane-associated. The different quaternary assemblies of the four TFE subfamilies and the electrostatic surface features between the active sites, as well as membrane association, have been speculated to play a role in the possible mechanisms of substrate channeling between the active sites of these TFEs (Ishikawa *et al.*, 2004 ▸; Sah-Teli *et al.*, 2020 ▸, 2023 ▸). The reaction intermediates of the three TFE reactions are always negatively charged due to the phosphate groups of the CoA moiety, and it has been proposed that positively charged residues on the surfaces of these proteins are used to guide the negatively charged intermediates between the three active sites (Venkatesan & Wierenga, 2013 ▸). In addition to the charged surfaces, the formation of reaction chambers, due to the mode of assembly, has also been proposed to play a role in substrate channeling. For HsTFE and anEcTFE, membrane association is also hypothesized to be important in this respect (Sah-Teli *et al.*, 2023 ▸; Xia *et al.*, 2019 ▸).

The crystal structure of MtTFE in its unliganded and CoA-bound forms identified the binding sites for its CoA-conjugated substrates to be at each of its three active sites [CoA(ECH), CoA(HAD) and CoA(KAT)] (Venkatesan & Wierenga, 2013 ▸), with the binding site for the NAD^+^ cofactor being at the HAD active site (Dalwani *et al.*, 2021 ▸). A structural analysis of the MtTFE structure highlighted the presence of positively charged residues between the ECH and HAD active sites and a neutral surface path between the HAD and KAT active sites (Dalwani *et al.*, 2021 ▸; Venkatesan & Wierenga, 2013 ▸). These crystallographic binding studies also revealed three additional CoA binding sites on the surface of MtTFE, referred to as the CoA-B(ECH2), CoA-A(HAD/KAT) and CoA-C(ECH/HAD) sites (Dalwani *et al.*, 2021 ▸; Fig. 1 ▸).

Crystallographic fragment screening has been established as an attractive and useful tool to identify regions on the surface of proteins to which small-molecule ligands (molecular mass of <250 Da) bind with weak affinity (Martin & Noble, 2022 ▸; Wollenhaupt *et al.*, 2020 ▸). These studies have shown that functional binding sites can be identified by fragment-binding studies (Carbery *et al.*, 2022 ▸; Radeva *et al.*, 2016 ▸), even if the affinity for the fragments is low and difficult to detect using other biophysical methods (Price *et al.*, 2017 ▸; Schiebel *et al.*, 2016 ▸). Further studies are required to establish the key features of the binding determinants at the surface patches that are identified in the fragment-screening experiments (Anderson, 2022 ▸; Czub *et al.*, 2022 ▸; Davies *et al.*, 2011 ▸; Hilario *et al.*, 2016 ▸). The information obtained from a crystallographic fragment-binding study has also been used in the early stages of drug-discovery research, in particular by combining the structural information from multiple fragment hits to generate larger lead compounds with higher affinity, which has subsequently led to the development of new potential drug molecules (Boby *et al.*, 2023 ▸; Erlanson *et al.*, 2016 ▸; Heightman *et al.*, 2018 ▸). New functional binding sites have been identified using this approach (Krojer *et al.*, 2020 ▸; Shumilin *et al.*, 2012 ▸; Skaist Mehlman *et al.*, 2023 ▸). Here, fragment screening has been used as a tool (i) to identify low-affinity binding pockets on the surface of MtTFE related to the mode of binding of the acyl tails of the acyl-CoA substrates, as well as (ii) to find binding regions of relevance for substrate channeling of reaction intermediates between these three active sites.

## 2. Methods

### 2.1. Protein expression and purification

Recombinant MtTFE was produced in *E. coli* BL21 (DE3) cells and purified using a previously standardized protocol (Venkatesan & Wierenga, 2013 ▸). Once purified, the enzyme was concentrated in storage buffer [20 m*M* 4-(2-hydroxy­ethyl)-1-piperazineethanesulfonic acid (HEPES–NaOH) pH 7.2, 120 m*M* KCl, 1 m*M* EDTA, 1 m*M* NaN_3_], flash-frozen using liquid nitrogen and stored at −70°C for further use.

### 2.2. Protein crystallization

MtTFE was crystallized in its unliganded form using a previously described protocol (Venkatesan & Wierenga, 2013 ▸). For this, 0.5 µl MtTFE solution (6 mg ml^−1^ in storage buffer) was mixed with crystallization well solution consisting of 2 *M* ammonium sulfate in 100 m*M* tris(hydroxymethyl)aminomethane (Tris–HCl) buffer pH 8.5 in a 1:1 ratio using a Mosquito nanodispensing robot (TTP Labtech) and crystallized by vapor diffusion in hanging drops (total volume 1 µl) at room temperature. The plates were imaged using a Formulatrix Rock Imager (RI54) at regular time intervals and the formation of the crystals was monitored using the *IceBear* software (Daniel *et al.*, 2021 ▸).

### 2.3. Choice of fragment libraries and preparation of the fragment-containing drops

#### 2.3.1. Library from Helmholtz-Zentrum Berlin (HZB)

96 fragments selected for general crystallographic fragment-screening purposes based on size, diversity, presence as a ligand in other PDB entries, cost and availability (Huschmann *et al.*, 2016 ▸) were pre-spotted in a single fragment per spot format as provided by HZB. 100 nl MtTFE crystallization solution (2 *M* ammonium sulfate, 100 m*M* Tris–HCl pH 8.5) was pipetted onto each deposited fragment spot such that completely soluble fragments would be at a final concentration of 100 m*M* in the fragment drop. Subsequently, 50 µl of the same MtTFE crystallization solution was immediately pipetted as the reservoir solution into the wells of all of the spotted fragments, the plate was sealed and the fragment drops were allowed to equilibrate against the crystallization solution for 24 h at room temperature before starting the crystal-soaking experiment.

#### 2.3.2. Compounds from Philipps-University Marburg

In order to mimic the negatively charged acyl-CoA substrates/intermediates of MtTFE, 130 negatively charged small-molecule compounds were provided as pre-weighed powder in tubes or as an aqueous solution. A 1.0, 0.5 or 0.25 *M* stock concentration of each of the provided compounds was made by dissolving the powder in either 100% DMSO or 50%(*v*/*v*) DMSO/water. The four compounds provided as aqueous solutions were used as specified in Supplementary Table S1. Not all of the compounds dissolved completely when the calculated volume of solvent was added. In these cases, the suspension of the compound was vortexed vigorously before being used for spotting. For the spotting, 100, 200 or 400 nl of each fragment suspension was deposited onto a TTP Labtech plate with a single fragment per deposited spot. The drops were dried off at room temperature (20°C) or in an incubator at 25°C for up to 48 h until no solvent was visible. Once the solvent had completely evaporated, 70 µl MtTFE crystallization solution (2 *M* ammonium sulfate, 100 m*M* Tris–HCl pH 8.5) was pipetted into the wells of the TTP Labtech plate and the spotted ligands were reconstituted with 500 nl of the same well solution using a Mosquito nanodispensing robot such that the nominal concentration of each of the compounds was 200 m*M*. The drops were sealed and subsequently allowed to equilibrate for 24 h at room temperature before starting the crystal-soaking experiment.

### 2.4. Fragment soaking

For fragment soaking, the individual soaking experimental approach was adopted, in which 5–10 unliganded crystals of MtTFE were transferred into each of the 226 pre-spotted fragment-containing drops and incubated at room temperature for at least 24 h before cryocooling in liquid nitrogen. The suitability of the MtTFE crystals for the fragment-soaking experiments was assessed by the visual inspection of images obtained from *IceBear* (Daniel *et al.*, 2021 ▸). Mostly larger crystals (larger than 100 µm in diameter) that had grown in drops with a single or a few crystals per drop were selected. Once selected, crystals were harvested and manually transferred using a loop into the sitting drop that contained the pre-spotted fragment. Subsequently, the crystals were allowed to equilibrate in the fragment solution for 24–48 h at room temperature, directly cryocooled in liquid nitrogen and subsequently stored in liquid nitrogen for the diffraction experiment. In some cases the crystals cracked or dissolved during the soaking step before they could be cooled, and in some cases crystals that survived the soaking step diffracted to less than 3.2 Å resolution. Thus, these could not be checked further for binding studies and were excluded from the data-processing and analysis steps.

### 2.5. Data collection, data processing and structure refinement

Frozen fragment-soaked crystals were transferred in dry shippers to various European synchrotrons for data collection. X-ray diffraction data sets were collected on different beamlines at BESSY II, DLS, MAX IV and PETRA III. All data collection was performed at a temperature of 100 K. Data reduction was either performed manually using *XDS* (Kabsch, 2010 ▸) and *AIMLESS* (Evans & Murshudov, 2013 ▸) or by using the various data-processing pipelines available at the different synchrotron facilities, including *XDSAPP* (Sparta *et al.*, 2016 ▸; Krug *et al.*, 2012 ▸), *xia*2 (Winter, 2010 ▸), *xia*2_3*dii* (Winter, 2010 ▸), *fast_dp* (Winter & McAuley, 2011 ▸), *xia*2_*DIALS* (Winter *et al.*, 2018 ▸), *autoPROC* (Vonrhein *et al.*, 2011 ▸) and *STARANISO* (Tickle *et al.*, 2016 ▸). Only data sets that could be processed to a resolution of 3.2 Å or better were used for molecular-replacement calculations to obtain initial phases. As a criterion for the resolution limit, the CC_1/2_, *I*/σ(*I*) and completeness were monitored. The data-collection and data-processing statistics are listed in Supplementary Tables S2 and S3. The model used as a search model in molecular replacement was derived from PDB entry 4b3h, after removing bound ligands and water molecules, using either *Phaser* (McCoy *et al.*, 2007 ▸) or *MOLREP* (Vagin & Teplyakov, 2010 ▸). The positioned coordinates after molecular replacement were used as the initial model to perform iterative rounds of model building and refinement using *Coot* (Emsley *et al.*, 2010 ▸), *phenix.refine* from *Phenix* (Afonine *et al.*, 2012 ▸; Liebschner *et al.*, 2019 ▸) and *REFMAC*5 from *CCP*4 (Murshudov *et al.*, 2011 ▸; Potterton *et al.*, 2018 ▸; Agirre *et al.*, 2023 ▸). Ligands were built into their electron density only after several rounds of manual model building and refinement and after adding sulfate ions and water molecules. Disordered side chains were included in the model, but not always at the beginning and end of the built protein chains. In a few cases side chains were built in double conformations. The obtained refined structures of the MtTFE–fragment complexes were subsequently analyzed with *PDB-REDO* (Joosten *et al.*, 2011 ▸), in particular with regard to the protein model and the water structure. Refinement of all structures was completed using *phenix.refine*. The structure quality was assessed using *MolProbity* (Williams *et al.*, 2018 ▸) as well as by inspecting the validation report from the PDB validation server (Feng *et al.*, 2021 ▸; Smart *et al.*, 2018*b* ▸). Three regions of the protein part of the structure were difficult to build in several structures: domain C of the α-subunit (in particular in chain *A*, because of the few crystal contacts), residues 570–580 of the α-subunit (in particular in chain *B*) and residues 225–231 of both thiolase β-subunits (referred to as the KAT225 loop). Domain C is the somewhat flexible domain of the HAD active site. The region 570–580 of the α-subunit is not near any of the active sites. In some structures this loop was not completely built. As discussed in Section 3, the KAT225 region of the β-chain is of functional relevance; it adopts either a helical conformation (as seen in structures with CoA bound in the thiolase active site; PDB entry 7o4t) or a looped-out conformation (as seen in the unliganded structure; PDB entry 7o4q), and in some structures it is disordered and was not built. The C^β1^–C^α1^ region of the thiolase subunit, which contributes to the shape of its acyl-tail binding pocket, has high *B* factors (Dalwani *et al.*, 2021 ▸) and this region was not completely built in the structure of the M-80 complex.

The quality of fit between the modeled fragment and the observed electron-density map was assessed by manually checking the electron-density maps as well as by using the real-space correlation coefficient (RSCC) of the bound fragment (as provided in the PDB validation report; Smart *et al.*, 2018*a* ▸). An RSCC value of 0.8 was chosen for accepting bound fragments, with a few exceptions in cases where fragments were found to be present in both copies of the asymmetric unit or where the binding of another similar fragment at the same binding site was observed. In addition, omit *mF*_o_ − *DF*_c_ difference maps, obtained from *PDB-REDO* refinement calculations using models in which the fragments were omitted, were inspected. A bound fragment was only retained if positive electron-density features corresponding to the bound fragment were present in these unbiased omit *mF*_o_ − *DF*_c_ difference maps. Occupancies were not refined, but in some cases the occupancies were manually set from the information in the difference maps (Supplementary Table S4). The final refinement statistics are listed in Supplementary Tables S2 and S3. A summary of the data-processing and refinement statistics of the refined structures is provided in Table 1 ▸. The unbiased omit *mF*_o_ − *DF*_c_ difference maps (Tables 2 ▸ and 3 ▸, Supplementary Table S1) were calculated after refinement using *phenix.refine* of a model in which the relevant ligand had been deleted.

### 2.6. Ligand restraints

The restraints for the fragments were generated according to the structural formulae provided in Supplementary Table S1. Coordinates and geometry restraints for the HZB compounds were provided by HZB. Coordinates and restraints for the set of compounds that were provided by Philipps-University Marburg were generated from SMILES strings using the *GRADE* web server (Smart *et al.*, 2011 ▸). For fragment M-1 (PDB Chemical Component ID A9J), the *REEL* software from *Phenix* (Moriarty *et al.*, 2017 ▸) was used to fix the ligand geometry in the restraints file available from the PDB. For fragment M-72 the electron-density maps showed the mode of binding of 11 fragments. In six binding events it involved molecules with the structure as provided in Supplementary Table S1 (PDB Chemical Component ID JXL). In two cases a dimeric derivative (Supplementary Fig. S1) was observed (PDB Chemical Component ID YLN) [covalently bound to the side chain of His(−9) of the *A* and *B* chains] and in three other binding events either the monomeric form (two binding events) or the dimeric form (one binding event) were suggested by the electron-density map to be modified, which was modeled as a partially ordered glycerol moiety (PDB Chemical Component IDs YLZ and YMK, respectively). The geometries of these modified fragments are in agreement with known boron chemistry (Diaz & Yudin, 2017 ▸). The restraints of the covalent link between the dimer fragment and the histidine side chain were generated using *JLigand* from *CCP*4 (Nicholls *et al.*, 2021 ▸). In the M-10 structure the electron-density map also suggested (in two cases) a modification which was modeled as a dimeric derivative of the used fragment (Supplementary Fig. S1).

### 2.7. Mass spectrometry

Fragments M-80, M-83, M-92 and M-109 were provided as aqueous solutions created through hydrolysis of the corresponding sulfonyl chlorides by dissolving them in 1 *M* aqueous NaOH. The conversion of these sulfonyl chlorides to the respective sulfonic acids (Supplementary Table S1) was checked using electrospray ionization mass spectrometry (ESI-MS). In addition, the oligomeric state of fragment M-72 was also checked using ESI-MS, due to its exhibiting the formation of a dimeric species in some binding events of this compound in the electron-density maps of the crystal structures. Each fragment was diluted 100-fold from the initial stock solution into 50% methanol/50% water. The diluted fragment solution was directly injected at a rate of 5 µl min^−1^ into a Q-Exactive Plus Mass Spectrometer (Thermo Scientific) and measured in negative mode. Raw data were analyzed using *X-Calibur Qual Browser* (Thermo Scientific) to identify the compounds of interest by their accurate mass. The mass-spectrometric analysis confirmed that the samples of fragments M-80, M-83, M-92 and M-109 contained the respective sulfonic acids, whereas the corresponding sulfonyl chlorides were not detected, and that the M-72 sample also contained a dimeric derivative of fragment M-72 (Supplementary Fig. S1).

### 2.8. Structure analysis

The mode of binding of CoA at the CoA-A(HAD/KAT), CoA-B(ECH2) and CoA-C(ECH/HAD) binding sites is provided by the coordinates of PDB entries 7o4r (2.8 Å resolution, referred to as the CoA-A structure), 7o4s (2.8 Å resolution, referred to as the CoA-B structure) and 7o4t (2.1 Å resolution, referred to as the CoA-C structure), respectively (Dalwani *et al.*, 2021 ▸). The mode of binding of CoA in the ECH and KAT active sites is also provided by these structures, and the CoA-C structure (PDB entry 7o4t) is used as the reference coordinate set. The reference structure for the unliganded complex is PDB entry 7o4q. The electrostatic potential at the molecular surface of the MtTFE tetramer was calculated and visualized using *CCP*4*MG* (McNicholas *et al.*, 2011 ▸) using the CoA-C structure (PDB entry 7o4t) as the model. In this structure, the main chain of all four chains is completely built. To generate the model for the electrostatic surface calculations, all of the ligand and water molecules were first deleted, and the disordered missing side chains of arginine, lysine, glutamate and aspartate residues were then modeled into the structure. The structures of the MtTFE–fragment complexes were superimposed onto the CoA-C structure using the *SSM* tool (Krissinel & Henrick, 2004 ▸) in *Coot* and the fragment binding with respect to the electrostatic surface was also visualized using *CCP*4*MG* (McNicholas *et al.*, 2011 ▸). Supplementary Movie S1 was generated using *CCP*4*MG* (McNicholas *et al.*, 2011 ▸). All structural figures were generated using *PyMOL* (version 2.0; Schrödinger).

## 3. Results and discussion

### 3.1. The 16 structures of MtTFE–fragment complexes identify new binding sites on the surface of MtTFE

Crystallographic fragment screening of a total of 226 fragments was performed for MtTFE using two compound libraries: (i) a library of 96 chemically diverse small molecules from HZB and (ii) a collection of 130 small molecules obtained from the University of Marburg, selected to include a negative charge, as also present in the substrates and reaction intermediates of MtTFE. Of the 226 fragments that were tested, 19 hits, *i.e.* MtTFE structures with bound fragments, were obtained, 14 of which belonged to the Marburg collection, indicating that the choice of a library that has more negatively charged fragments was more successful for MtTFE. For three of the 19 structures, the features of the electron-density map at the ligand-binding sites were insufficient to satisfactorily decide the ligand orientation. For these three structures, the fragment-binding sites overlapped with sites that were also identified in the structures of other MtTFE–fragment complexes; therefore, these three structures were excluded from further refinement and the structures of 16 fragment-binding experiments (five HZB fragments and 11 Marburg fragments) were selected and used for further analysis (Table 1 ▸, Supplementary Movie S1). Of these, the structures of 12 fragments were refined to a resolution of 2.8 Å or better, two fragments to 2.9 Å resolution and two fragments to low resolutions of 3.0 and 3.2 Å, respectively. Unlike typical fragment-screening campaigns that are carried out to identify the precise binding modes of fragments as starting points for the development of lead candidates for drug-discovery purposes, our fragment-screening experiments were aimed at identifying low-affinity binding sites on the surface of MtTFE. Therefore, data sets of somewhat lower resolution are also informative and these low-resolution structures were also retained for further analysis. Key structure-refinement statistics are provided in Supplementary Tables S2 and S3. All of the built fragments have a good fit to the electron-density map, as described in Section 2 (their RSCC values are listed in Supplementary Table S4). Representative omit *mF*_o_ − *DF*_c_ difference maps are shown in Tables 2 ▸ and 3 ▸ and Supplementary Table S1.

**Figure 2:**
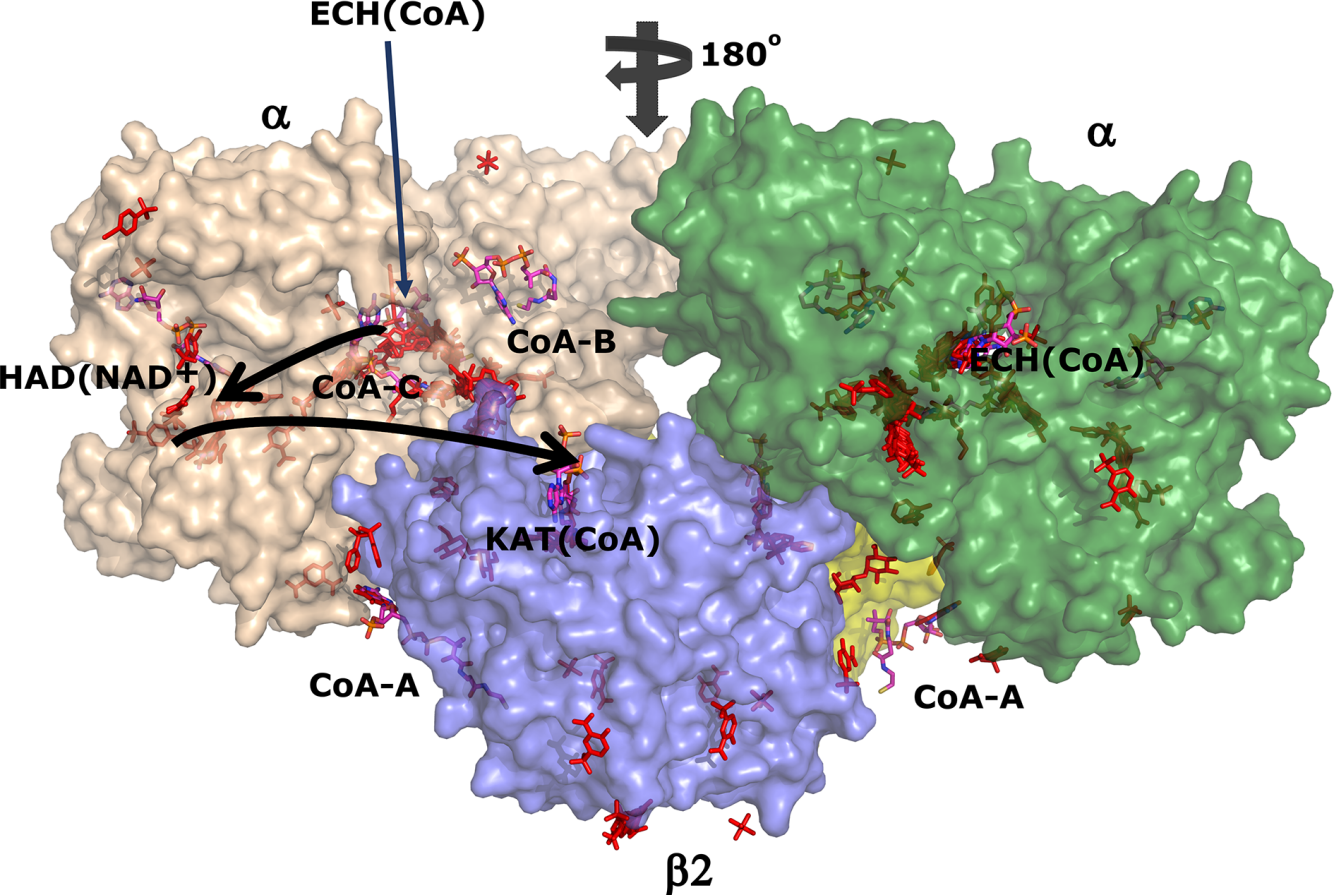
The 121 binding events on the surface of the TFE tetramer. The same view and coloring scheme are used as in Fig. 1 ▸. The two α-subunits are colored wheat and green and the β_2_ thiolase dimer subunits are colored cyan and yellow. The ECH, HAD and KAT active sites are identified as ECH(CoA) (with a thin arrow), HAD(NAD^+^) and KAT(CoA). The vertical arrow visualizes the twofold axis of the α_2_β_2_ tetramer. The ECH active site of the left α-subunit (wheat) is behind and the ECH active site of the right α-subunit (green) is at the front, showing the fragments bound in its substrate-binding tunnel. The surface has been made transparent, so that binding events that are hidden behind the surface are still visible. The labels CoA-A, CoA-B and CoA-C identify the additional CoA binding sites.

The 16 fragments are bound over a total of 121 individual binding events. In seven binding events double conformations are observed (Supplementary Table S4), in two cases because the binding event is on a crystallographic twofold. The 121 binding events overlap to a great extent with the previously identified CoA binding sites at the ECH, HAD and KAT active sites as well as with the CoA-A(HAD/KAT), CoA-B(ECH2) and CoA-C(ECH/HAD) binding sites (Table 4 ▸). Most fragments bind at multiple binding sites (Table 5 ▸), for example fragment M-1 (25 binding events) and fragment M-76 (14 binding events). In most instances, binding at each site is observed in both copies of the αβ dimer of the MtTFE tetramer. 94 out of 121 binding events (14 out of 16 fragments) have an aromatic moiety and 107 out of 121 binding events involve negatively charged fragments (11 out of 16 fragments, eight of which contain a sulfonic acid group) (Supplementary Table S1). Although the mother liquor of the crystals used in these experiments contains 2 *M* ammonium sulfate and there are approximately 25 sulfate molecules bound at various surface sites in each structure, the sulfonic acid-containing fragments as well as the M-1 fragment (the 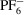 anion) do not bind at these sulfate-binding sites. A total of 88 fragment-binding events (73%) occur at one of the previously identified binding sites, namely the three active sites (including the NAD binding pocket), or any of the three additional CoA binding sites or in associated pockets near the bound CoA or NAD^+^ molecules (Table 5 ▸). Of these 88, only two binding events occur at a crystal contact (fragment M-80, bound at subsite H0). 33 individual fragments (27%) bind at previously unidentified binding sites scattered over the surface of the tetramer (Fig. 2 ▸); 12 of these sites are at crystal contacts. 41 fragment-binding events occur at the ECH active site (Table 5 ▸), which is the maximum number of hits at a single binding pocket, indicating that this is the fragment-binding ‘hotspot’ of MtTFE.

It is noteworthy that in some structures blobs of positive difference electron density could not be assigned to the soaked fragments and in these cases the densities have been left unmodelled. This may be, for example, because of partial disorder of the bound fragment. One fragment (M-72) was observed in its unmodified monomeric form (see, for example, Table 2 ▸), but the electron-density map also showed it to be present in a dimeric form (Supplementary Fig. S1) as well in modified monomeric and dimeric forms. The modification of the monomeric form was modeled as a partially disordered glycerol adduct, although the precise nature of the modification is not known. This modification was also observed for one binding event of the dimeric form. In two binding events this dimeric form had reacted with a histidine side chain of the enzyme. The modified histidine side chain is observed in both copies of the N-terminal His tag of the α-chain, at His(−9), and its binding site is at the exit of the substrate-binding tunnel of the ECH active site. The structures of the modeled derivatives of M-72 are in agreement with the known structural properties of this boron compound (Diaz & Yudin, 2017 ▸). The occurrence of the monomeric and dimeric forms of M-72 in the stock solution was verified by mass spectrometry (see Section 2). The bound unmodified M-72 molecules and the modified M-72 derivatives included in the final structure have a good fit to the electron-density map, with RSCC values of 0.81 or higher. None of the experiments with the other compounds resulted in a covalent modification of MtTFE. For one other fragment (M-10), the electron-density maps also suggested that the fragment was modified in two binding events, being a dimeric form (Supplementary Fig. S1).

### 3.2. The fragment-binding events that are mapped to the 15 subsites

Previous crystallographic binding studies of MtTFE with CoA identified the ECH, HAD and KAT active sites, as well as three additional CoA binding sites referred to as theCoA-A(HAD/KAT), CoA-B(ECH2) and CoA-C(ECH/HAD) binding sites based on their location on the surface of the MtTFE tetramer (Dalwani *et al.*, 2021 ▸; Table 4 ▸, Fig. 1 ▸). Each of these six sites occurs in pairs, as the asymmetric unit of this crystal form is the α_2_β_2_ tetramer, in which a local twofold axis (Fig. 1 ▸) relates the two αβ dimers to each other. Many binding sites of the fragments overlap with these CoA binding regions, but also identify binding pockets near these CoA binding sites; for example, regions where the acyl tail of the acyl-CoA substrate molecules could bind, as discussed further below. The binding pockets at the active sites for the acyl tail of the acyl-CoA substrates have been predicted in previous studies (Dalwani *et al.*, 2021 ▸).

The substrates of MtTFE are large molecules (molecular mass of >900 Da), larger than the bound fragments, and therefore each of the active-site CoA binding pockets are described in terms of smaller subsites from the pantetheine region (site-1), via the catalytic site (site-2), to the acyl-tail region (site-3), as defined in Table 4 ▸: (i) three subsites at the ECH active site (E1, E2 and E3), (ii) four subsites at the HAD active site [H0 (the NAD binding pocket), H1, H2 and H3] and (iii) three subsites at the KAT active site (K1, K2 and K3).

The CoA-C(ECH/HAD) additional CoA binding site and its nearby regions are also divided into three subsites (I1, I2 and I3, as defined in Table 4 ▸). Only a few fragments are bound at the other two additional CoA binding sites and therefore these sites are referred to as the C1 and C2 sites for the CoA-A(HAD/KAT) and CoA-B(ECH2) sites, respectively. In this way, 88 fragment-binding events are mapped to these 15 subsites (Table 5 ▸). In addition, 33 other binding events are observed at binding sites scattered over the surface of the MtTFE tetramer (Fig. 2 ▸, Table 5 ▸). In the next sections, the fragment binding in the three active sites is discussed and the binding events at the three additional CoA binding sites are subsequently described.

### 3.3. Fragment binding at the three active sites includes the proposed CoA acyl-tail binding pockets

Previous crystallographic binding experiments of seven MtTFE active-site point-mutated variants with 2*E*-enoyl-CoA substrates did not yield structures of complexes of MtTFE with bound acyl-CoA substrate/intermediate molecules, but instead resulted in CoA-bound structures (Dalwani *et al.*, 2021 ▸). Thus, although these previous studies identify the binding sites for the CoA moiety of the acyl-CoA substrate at each of the active sites, as well as the binding of CoA at three additional sites, these studies did not provide experimental evidence for the mode of binding of the acyl-tail part of the acyl-CoA substrate in any of the active sites. However, in addition to fragment binding being observed at the CoA binding sites, the current fragment-screening experiments also resulted in MtTFE structures in which fragments are bound at the predicted acyl-tail binding pockets of each of the active sites, referred to in Table 4 ▸ as E3, H3 and K3, as discussed further in the next sections. A similar overlap of the binding sites for fragments and fatty acids has also been described in recent crystallographic binding studies of serum albumins with ketoprofen (Anderson, 2022 ▸; Czub *et al.*, 2022 ▸) using a fragment with similar properties as used in these MtTFE studies (ketoprofen has a negative charge and two aromatic rings).

### 3.4. The ECH active-site binding pocket

**Figure 3:**
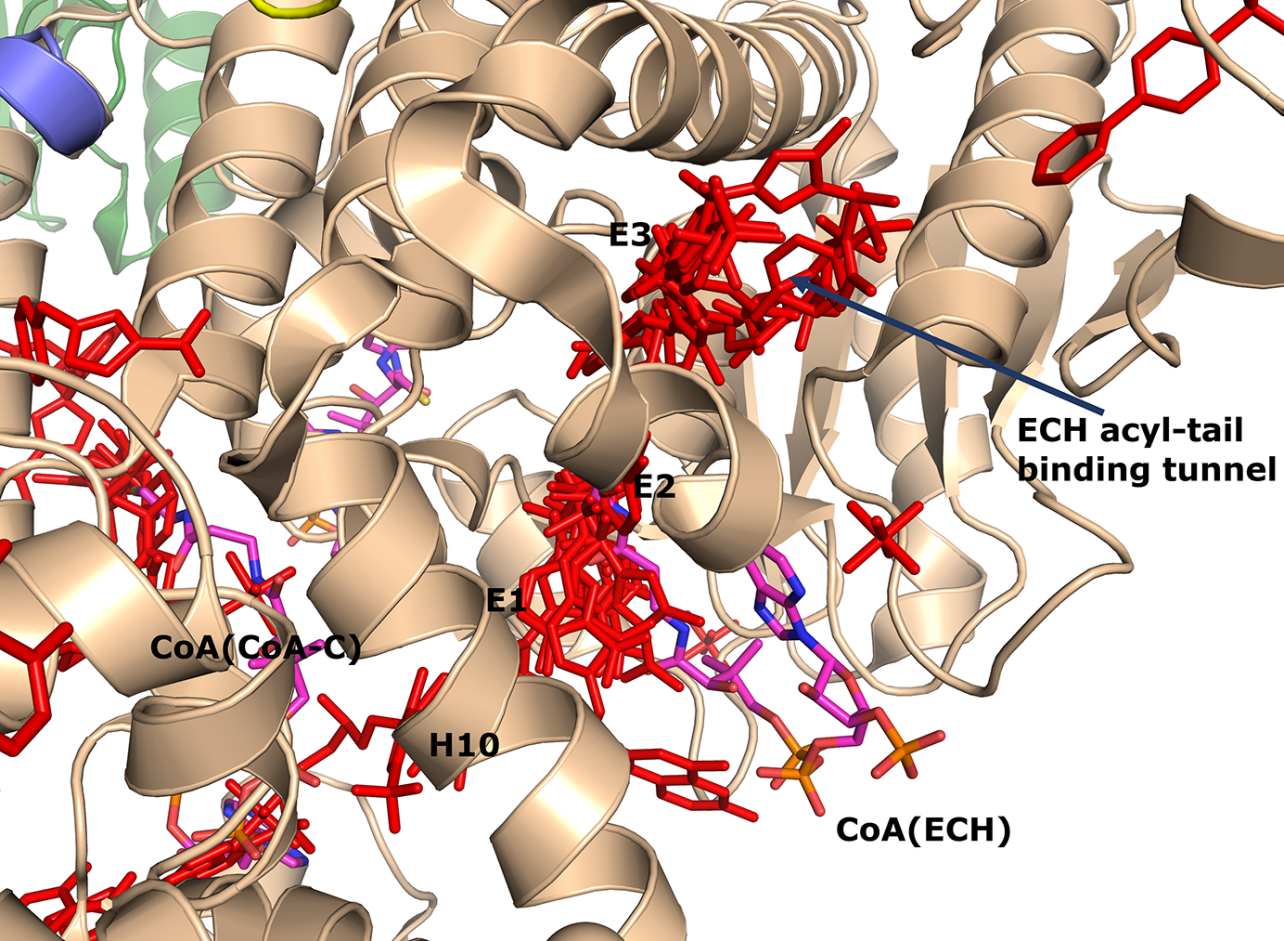
Fragment-binding events at the ECH active site. View into the ECH acyl-tail binding tunnel. The C-terminal helix of the crotonase fold (helix H10) covers the ECH active site. Also included are CoA as bound to the ECH active site [magenta, labeled CoA(ECH)], as well as the CoA bound in the CoA-C(ECH/HAD) site [magenta, labeled CoA(CoA-C)]. The bound fragments cover the active-site pocket (E1, E2 and E3) and extend to the exit of the ECH acyl-tail binding tunnel.

**Figure 4:**
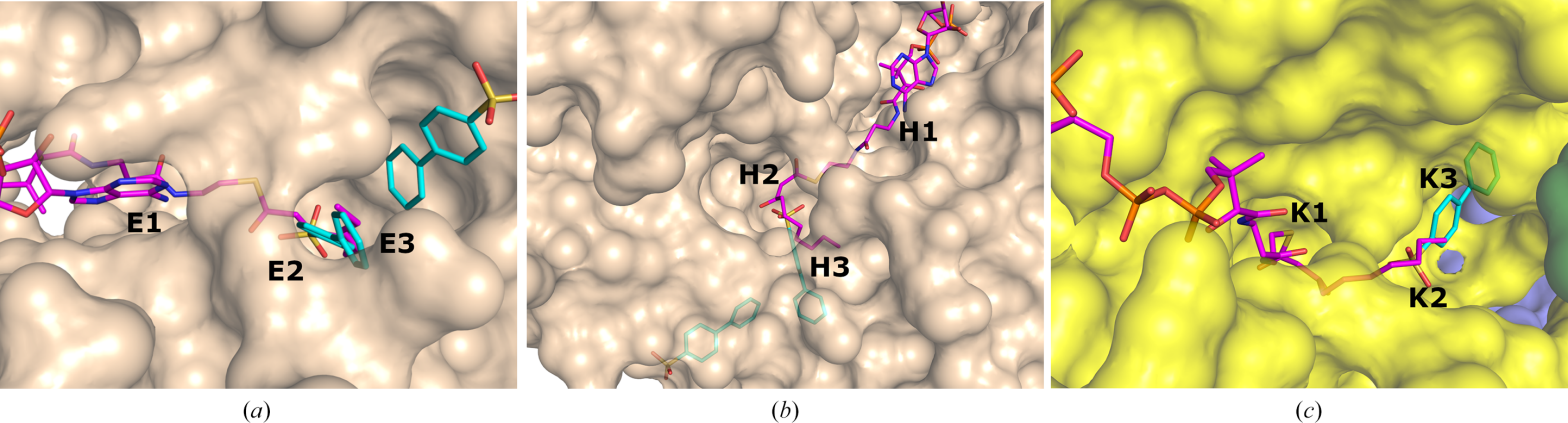
The acyl-tail binding pocket of the ECH (*a*), HAD (*b*) and KAT (*c*) active sites. The mode of binding of fragment M-83 (cyan) to the subsites is shown, together with the superimposed mode of binding of the acyl-CoA substrate (magenta), as predicted by model building (Dalwani *et al.*, 2021 ▸). The predicted mode of binding of the acyl tail of the substrates overlaps with the fragments bound at regions E2 and E3 (residues A811 and A812), at H2 and H3 (residues A813 and A809) and at K2 and K3 (residue C508) of the three active sites. The omit *mF*_o_ − *DF*_c_ difference maps of these residues are shown in Table 2 ▸. Interactions of these fragments with active-site residues are visualized in Supplementary Fig. S2.

A majority of the fragments (12 out of 16 fragments, 41 binding events) bind in the ECH active site, making it the fragment-binding hotspot of our fragment-screening experiments (Fig. 3 ▸, Table 5 ▸). These fragments are bound at multiple sites across the entire ECH active site. The mode of binding of fragment M-83 in the ECH active site is shown in Fig. 4 ▸. Its sulfonate group binds in the active site, near the catalytic glutamates Gluα119 and Gluα141 (Supplementary Fig. S2). A similar mode of binding is observed for fragment B-H11, which interacts with these two catalytic glutamates using its hydroxyl group. The seven fragments M-10, M-49, M-76, M-79, M-80, M-92 and M-109 share a common substructure of a sulfonic acid functional group connected to an aromatic ring. The sulfonate groups of these seven fragments bind in the same region of the ECH catalytic site, but the aromatic group is bound in two different ways, such that for M-10, M-49, M-76, M-79 and M-109 it overlaps with the mode of binding of the pantetheine moiety of the substrate, whereas for M-80 and M-92 it points towards the E3 region. Altogether, the bound fragment molecules cover the entire ECH active site from the pantetheine binding site through the acyl-tail binding tunnel to the exit of this tunnel (Fig. 3 ▸). The bound fragment molecules thereby also overlap nicely with the predicted mode of binding of the acyl tail of 2*E*-decenoyl-CoA from previous modeling experiments (Dalwani *et al.*, 2021 ▸), as shown in Fig. 4 ▸ using the bound M-83 fragments as an example. It is noteworthy that none of the fragments bind at the binding pocket for the adenine moiety of CoA.

### 3.5. The HAD active-site binding pocket

The substrate-binding groove of the HAD active site consists of the C domain (the NAD binding domain) and the D/E domains of the α subunit. The C domain is known to exhibit conformational flexibility with respect to the D/E domains such that it can exist in ‘open’ and ‘closed’ conformations. The fully closed conformation, competent for catalysis, is only captured in the structure of the complex of the homologous monofunctional human HAD with NAD^+^ and acetoacetyl-CoA (Barycki *et al.*, 2000 ▸). In the available structures of MtTFE, the substrate-binding groove of the HAD active site is always seen in its open conformation and domain C has high conformational flexibility (Dalwani *et al.*, 2021 ▸). Except for capturing NAD^+^ binding in the NAD binding pocket (subsite H0) of the HAD active site (Dalwani *et al.*, 2021 ▸), all previous crystallographic MtTFE binding experiments failed to capture any ligand binding in the 3*S*-hydroxyacyl-CoA binding pocket of the HAD active site (*i.e.* subsites H1, H2 and H3). In the current fragment-binding studies six fragments bind in the substrate-binding pocket and two fragments bind in the NAD binding pocket: M-1 and M-80 (Table 5 ▸). The M-1 binding event (of the hexafluoro­phosphate anion) is in the pocket which binds the adenine ring of NAD. The M-80 binding event is near a crystal-contact region and the mode of binding of the M-80 fragment is also stabilized by crystal contacts. The six fragments that bind in the substrate-binding pocket are B-E1, M-1, M-53, M-76, M-83 and M-109. Fragments B-E1 and M-83 form hydrogen bonds either to Serα512 or to both Serα441 and Serα512, both of which point into the catalytic site. These fragments also make weak interactions with the side chain of the catalytic Hisα462. The mode of binding of fragment M-83 is shown in Fig. 4 ▸ and Supplementary Fig. S2. Fewer fragments bind here compared with the ECH active site, but binding is observed in the same subsites as seen for the ECH active site, and the mode of binding of these fragment molecules also overlaps with the predicted mode of binding of 3-ketodecanoyl-CoA (Dalwani *et al.*, 2021 ▸). None of the bound fragments induces conformational changes that capture a closed conformation of the MtTFE HAD active site. Thus, in all structures of MtTFE reported thus far the C domain of the HAD active site is always seen in its open conformation

### 3.6. The KAT active-site binding pocket

The acyl-tail binding pocket of the KAT active site is the least accessible of the three active sites of MtTFE. The lowest number of fragments bind at the KAT active site (three out of 16 fragments bind in this active site and there are six binding events; Table 5 ▸). Each of these bound fragments (B-E1, M-1 and M-83) binds in the KAT acyl-tail binding tunnel, beyond the CoA sulfhydryl group (Fig. 4 ▸). B-E1 and M-83 interact weakly with the side chain of the catalytic cysteine Cysβ92. The *R*-SO_2_-*R* and *R*-SO_2_-OH moieties of B-E1 and M-83, respectively, bind at the same site, forming a hydrogen bond to the side chain of Glnβ149, as visualized in Supplementary Fig. S2 for the M-83 fragment. The five-membered ring of fragment B-E1 points away from the acyl-tail binding tunnel towards the KAT225 loop of the thiolase β-subunit (contacting the Leuβ228 side chain) and the H9A helix of the α-subunit (in particular Proα243). Leuβ228 and Proα243 line the groove between the I2 site and the K2 site. M-1 (the hexafluorophosphate anion) binds in the acyl-tail binding tunnel, displacing the side chain of Metβ134, which has moved. The bound fragment M-83 overlaps with the mode of binding of the acyl tail of 3-ketodecanoyl-CoA, as predicted from previous modeling experiments (Dalwani *et al.*, 2021 ▸) and shown in Fig. 4 ▸.

### 3.7. The fragment-binding events identify the CoA-C(ECH/HAD) site as a possible functional transient binding site

**Figure 5:**
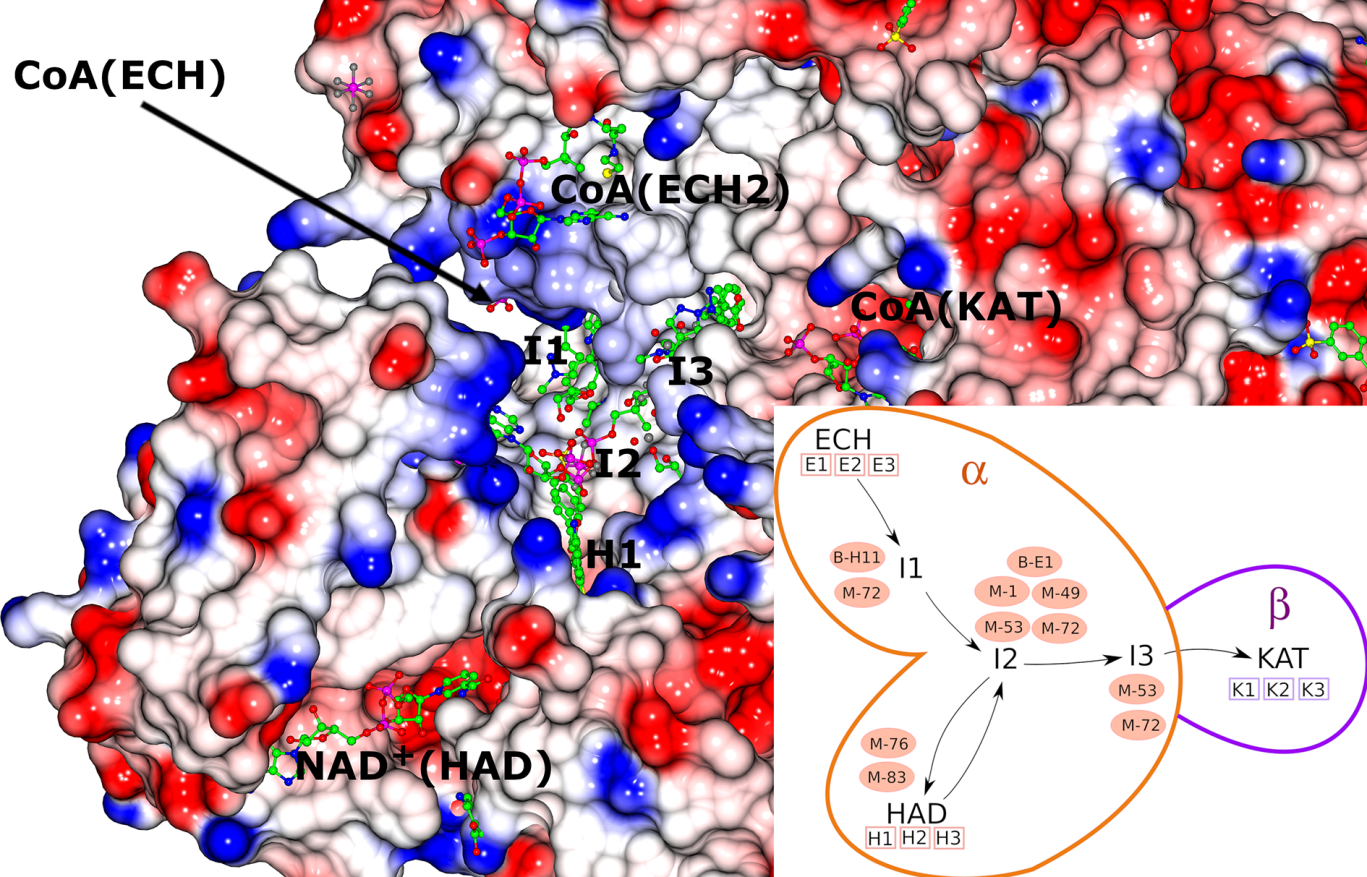
Properties of the protein surface of the possible substrate-channeling path between the three active sites of MtTFE. The image shows the color-coded molecular surface of the CoA-C(ECH/HAD) region, near the interface of chain *A* (α subunit) and chain *D* (β subunit) of the CoA-C structure (PDB entry 7o4t), color-coded such that the blue and red colors identify surface regions with positive and negative electrostatic potential, respectively. Neutral regions have a white color. The active sites are identified as CoA(ECH) (with an arrow), NAD^+^(HAD) (of the α subunit) and CoA(KAT) (of the β subunit). The CoA molecule bound at the CoA-C(ECH/HAD) site (I2) at the center of the surface between the three active sites is also included. I1, H1 and I3 identify the binding regions extending from I2 towards the ECH, HAD and KAT active sites, respectively. Also shown is CoA bound in the CoA-B(ECH2) site. A stereo figure is shown in Supplementary Fig. S3 and a video (Supplementary Movie S1) is also provided. The fragments included in the image are indicated in the inset, which schematically visualizes the locations of the I1, I2 and I3 sites with respect to the three catalytic sites in the same view as used for the molecular-surface image.

The reaction intermediates of MtTFE are polar negatively charged derivatives of CoA. Analysis of the surface features of MtTFE shows that its three active sites are separated by a surface path which is devoid of negatively charged residues (Fig. 5 ▸, Supplementary Fig. S3 and Supplementary Movie S1), and it has been proposed that this path could be used to transiently anchor the reaction intermediates, allowing them to crawl between the active sites, thus enabling substrate channeling (Venkatesan & Wierenga, 2013 ▸). Crystallographic binding studies with CoA have identified three additional CoA binding sites (Dalwani *et al.*, 2021 ▸), which to some extent overlap with this surface path. A comparison of the CoA–protein interactions of CoA bound at these additional binding sites versus the CoA–protein interactions of CoA bound in the active sites shows that on average the total number of atom–atom interactions per bound CoA (23.3 versus 27.5) and the total number of hydrogen-bond interactions (3.2 versus 8.5) are significantly lower for CoA bound in the additional binding sites compared with CoA bound in the active-site binding pockets (Supplementary Table S5). This is of particular interest for the CoA-C(ECH/HAD) binding site, which is located between the ECH, HAD and KAT active sites and which has been proposed to be part of the surface path relevant for substrate channeling (Dalwani *et al.*, 2021 ▸). At this binding site the CoA is bound in an extended conformation and the interactions between the bound CoA and the protein are much weaker than at the active sites (Supplementary Table S5). The location of this site and the weak CoA–protein interactions indicate that of the three additional CoA binding sites, the CoA-C(ECH/HAD) site could be a functional transient binding site for reaction intermediates.

The fragment-screening experiments identify multiple binding sites which overlap with the additional CoA binding sites. Seven out of 16 fragments (23 binding events) bind overlapping with at least one of the three additional CoA binding sites (Table 5 ▸). A single fragment (B-51) binds at the CoA-A(HAD/KAT) binding site (two binding events), one fragment (M-53) binds at the CoA-B(ECH2) binding site (one binding event) and five fragments (B-E1, M-1, M-49, M-53 and M-72) bind at the CoA-C(ECH/HAD) binding site (I2; 13 binding events). Clearly, the CoA-C(ECH/HAD) binding site is a favorable binding site for these fragments. Furthermore, near the CoA-C(ECH/HAD) site, fragments B-H11 and M-72 bind at subsite I1, which perfectly bridges the gap between the bound CoA at the ECH active site and the CoA-C(ECH/HAD) binding site. In addition, fragments M-76 and M-83 bind at H1 (the binding region of the pantetheine moiety of the HAD active site, which is close to I2) and fragments M-53 and M-72 bind at subsite I3, which extends beyond the CoA-C S atom towards the KAT active site. Fig. 5 ▸ shows the binding sites of these fragments in this region. The omit *mF*_o_ − *DF*_c_ difference maps of B-E1 (bound at I2), B-H11 (bound at I1), M-76 (bound at H1) and M-72 (bound at I3) are shown in Table 3 ▸. The 23 binding events at the H1, I1, I2 and I3 sites are defined by interactions with the α-subunit, except for the binding event of M-53 at the I3 subsite, which also involves interactions with the β-subunit (with βLeu231). Fig. 5 ▸ also provides a schematic visualization of the I1, H1 and I3 binding sites near the CoA-C(ECH/HAD) site (I2) with respect to the ECH, HAD and KAT active sites. The negatively charged functional group of fragment M-83 overlaps with the negatively charged pyrophosphate moiety of the CoA molecule bound at this site. Each of the fragments that bind in the CoA-C(ECH/HAD) binding region also binds to at least one of the three active sites (Table 5 ▸).

The proposed I3 binding groove between the CoA-C(ECH/HAD) site and the KAT active site is lined by residues α240–α250 of the α subunit (in particular Ileα241, helix H9A) and by residues Pheβ225-Glu-Gly-Leu-Ala-Ala-Leuβ231 just before the Lα5 helix of the thiolase subunit (in particular Leuβ231). The latter region, referred to as the KAT225 loop, is built as a high-*B*-factor loop in the unliganded structure of MtTFE (PDB entry 7o4q), but it adopts a helical conformation in the structures with CoA bound (for example, PDB entry 7o4t). This loop is an extension of the adenine binding loop (Harijan *et al.*, 2023 ▸), which has a conserved sequence fingerprint [Leu(β221)-Lys-Pro-Ala-Phe] that shapes the CoA binding pocket. In particular, Leuβ221 (pointing to the adenine ring) and Pheβ225 (pointing to the methyl groups of the pantetheine moiety), as well as Proβ223, are conserved in sequence alignments (Venkatesan & Wierenga, 2013 ▸; Harijan *et al.*, 2023 ▸). In some structures of the fragment complexes the KAT225 loop is disordered, in some structures it is built in a helical conformation (for example in the structure of the M-72 complex) and in some structures it is built in a loop conformation, such as for example in the structure of the M-53 complex. The binding pocket of one of the bound fragment M-53 molecules is shaped by the side chains of Ileα241 and Leuβ231. Further studies are required to understand the functional relevance of the different conformations of the KAT225 loop region.

### 3.8. The proposed substrate-channeling path identified by the fragment-binding events

As outlined above, in addition to identifying the CoA-C(ECH/HAD) site as a potential transient binding site (the I2 site) for reaction intermediates, these crystallographic fragment-screening experiments also identify subsites I1, H1 and I3, which are binding pockets that extend from the I2 CoA-C(ECH/HAD) binding site to the ECH, HAD and KAT catalytic sites, respectively. The crystallographic fragment-screening experiments therefore suggest a substrate-channeling path that could be used for the surface crawling of negatively charged reaction intermediates between the three active sites. The proposed path subsequently consists of the following binding sites: ECH (catalytic site) → I1 → I2 → H1 → HAD (catalytic site) → H1 → I2 → I3 → KAT (catalytic site). The distances from the ECH and HAD active sites to the I2 site are approximately 21 and 12 Å, respectively, and the corresponding distance from the KAT active site is approximately 44 Å, when considering the distances between the bridging pyrophosphate O atoms of the CoA molecules bound at these sites. The path is identified by the fragments B-E1, B-H11, M-1, M-49, M-53 and M-72, in addition to the CoA-C(ECH/HAD) molecule (Table 5 ▸, Fig. 5 ▸, Supplementary Fig. S3). Supplementary Movie S1 visualizes the results of these fragment-binding experiments, highlighting the proposed substrate-channeling path between the active sites, by showing the fragments as bound at sites along this surface path of MtTFE. The fragments M-1, M-49, M-53 and M-72 are negatively charged, like the MtTFE reaction intermediates. This path can be described as consisting of neutral residues, being lined by the side chains of positively charged residues (Lys/Arg; Fig. 5 ▸). Positively charged residues of the protein surface have been proposed to be important for steering the diffusion of negatively charged ligands for substrate channeling of the TS-DHFR bifunctional enzyme (Anderson, 2017 ▸; Metzger *et al.*, 2014 ▸) as well as in other biological systems (Zheng *et al.*, 2019 ▸). Other properties, such as disfavoring the binding of water molecules, referred to as ‘dewetting’ (Hilario *et al.*, 2016 ▸), have been described for the substrate-channeling path of the enzyme tryptophan synthase, in which a neutral indole molecule channels through a tunnel between its two active sites.

## 4. Concluding remarks

The crystal form of unliganded MtTFE used in these studies has several advantages in the identification of new, weak-affinity binding sites on the surface of MtTFE by the fragment-binding approach using crystal soaking. In this crystal form the asymmetric unit is the MtTFE tetramer, which means that there are two copies of each unique binding region per asymmetric unit. The percentage solvent in this crystal form is relatively high (*V*_M_ is 3.9 Å^3^ Da^−1^, which corresponds to 69% solvent), meaning that the molecules are loosely packed and therefore large portions of the surface of the protein are not involved in crystal-packing interactions, but instead can interact with solutes. Indeed, most binding events occur in pairs, being observed in both copies of the αβ dimer present in the asymmetric unit (Table 5 ▸), and only one binding event at the defined subsites (Table 4 ▸) is involved in crystal contacts. A drawback of these crystals is their relatively poor diffraction quality, routinely diffracting only to intermediate resolutions of between 2.2 and 2.8 Å, but lower resolution data sets have also been included. Typically, fragment-screening studies are carried out using crystals diffracting to better than 2 Å resolution, which allows a much more detailed study of the interactions between the fragment and protein. Nevertheless, analysis of the observed binding events provides an interesting insight into the fragment-binding properties of MtTFE, showing that the fragments used preferentially bind in the active sites, including in the predicted acyl-tail binding pockets (Fig. 4 ▸, Supplementary Fig. S2), as well as in regions between the active sites (Fig. 5 ▸). 14 (out of 16) of the characterized MtTFE–fragment complexes involve compounds with aromatic rings (Supplementary Table S1) [the only exceptions are fragment M-1 (the 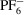 anion) and fragment B-B3 (trehalose)]. 11 (out of 16) fragments are negatively charged anions in solution. 65 fragment-binding events occur in pockets that shape the three active sites (Table 5 ▸), of which 28 involve the predicted binding pockets for the acyl tails. A very striking observation involves the 23 binding events in the region of the CoA-C binding site (I2), extending to the ECH, HAD and KAT active sites (I1, H1 and I3) (Fig. 5 ▸, Supplementary Fig. S3, Supplementary Movie S1). The visualization of the electrostatic surface properties indicates that this region is devoid of negative charges. The presence of a transient binding site (I2), extended by the binding sites I1, H1 and I3 to the ECH, HAD and KAT active sites, respectively, is consistent with a substrate-channeling mechanism of reaction intermediates between these active sites, as also described in binding studies of *Mtb* tryptophan synthase (Bosken *et al.*, 2022 ▸; D’Amico & Boehr, 2023 ▸) and the TS-DHFR bifunctional enzyme (Anderson, 2017 ▸; Metzger *et al.*, 2014 ▸). Thus, this MtTFE study provides a basis for further studies to better characterize the substrate-channeling properties of MtTFE. Orthogonal approaches would be required for such studies, in addition to these crystallographic binding experiments, to discover the acyl-CoA substrates for which channeling is relevant in MtTFE and also to validate the proposed path of channeling.

## 5. Related literature

The following references are cited in the supporting information for this article: Chen *et al.* (2010 ▸) and Incardona *et al.* (2009 ▸).

## Supplementary Material

PDB reference: *M. tuberculosis* trifunctional enzyme, complex with B-E1, 8opu

PDB reference: complex with B-H11, 8opv

PDB reference: complex with B-51, 8opw

PDB reference: complex with B-B3, 8opx

PDB reference: complex with B-77, 8opy

PDB reference: complex with M-1, 8oql

PDB reference: complex with M-10, 8oqm

PDB reference: complex with M-53, 8oqn

PDB reference: complex with M-49, 8oqo

PDB reference: complex with M-76, 8oqp

PDB reference: complex with M-79, 8oqq

PDB reference: complex with M-80, 8oqr

PDB reference: complex with M-83, 8oqs

PDB reference: complex with M-92, 8oqu

PDB reference: complex with M-109, 8oqv

PDB reference: complex with M-72, 8pf8

Supplementary Tables and Figures. DOI: 10.1107/S2059798324006557/ud5051sup1.pdf

Supplementary Movie S1. DOI: 10.1107/S2059798324006557/ud5051sup2.mp4

## Figures and Tables

**Table 1:** Summary of the refinement statistics for the structures of the 16 MtTFE–fragment complexes Detailed data-collection and refinement statistics are provided in Supplementary Tables S2 and S3. The covalent structures of the fragments are provided in Supplementary Table S1.

Data set	Resolution (Å)	*R*_work_ (%)	*R*_free_ (%)	R.m.s. bond-length deviation (Å)	PDB entry
B-E1	3.05	20.8	24.6	0.0016	8opu
B-H11	2.80	20.3	23.0	0.0017	8opv
B-51	2.52	20.5	24.0	0.0021	8opw
B-B3	2.90	18.9	22.4	0.0023	8opx
B-77	2.45	21.1	24.4	0.0025	8opy
M-1	2.7	18.4	21.9	0.0019	8oql
M-10	3.2	20.3	24.2	0.0016	8oqm
M-49	2.6	18.8	22.5	0.0017	8oqo
M-53	2.2	19.3	22.1	0.0021	8oqn
M-72	2.24	18.9	21.9	0.0021	8pf8
M-76	2.19	19.6	22.4	0.0025	8oqp
M-79	2.59	19.6	23.1	0.0020	8oqq
M-80	2.4	22.0	26.0	0.0024	8oqr
M-83	2.33	17.8	21.8	0.0033	8oqs
M-92	2.89	18.0	21.7	0.0019	8oqu
M-109	2.78	19.7	23.2	0.0019	8oqv

**Table 2:** Representative omit *mF*_o_ − *DF*_c_ difference maps of fragment-binding events at the three active sites as observed at the subsites defined in Table 4 ▸ The contour level of these maps is 2.5σ. Further information on the listed fragments is provided in Supplementary Table S1.

Binding site	Subsite	Residue	Omit *mF*_o_ − *DF*_c_ difference map
ECH	E1	M-72 (A809)	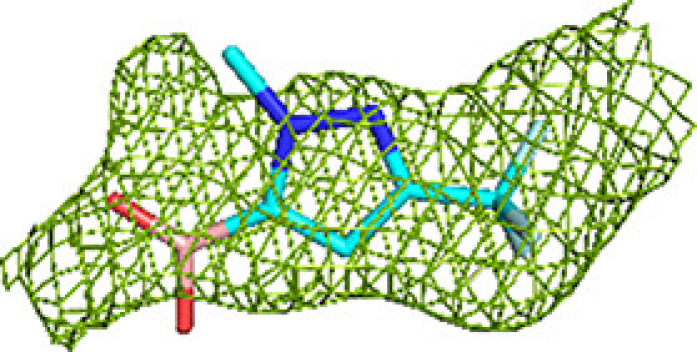
	E2	M-83 (A812)	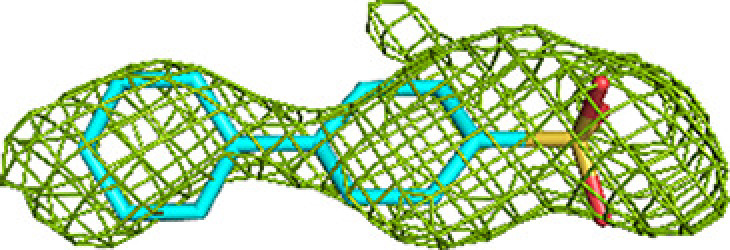
	E3	M-83 (A811)	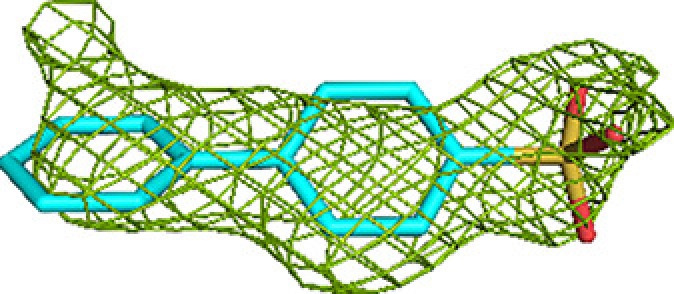
HAD	H0	M-1 (A814)	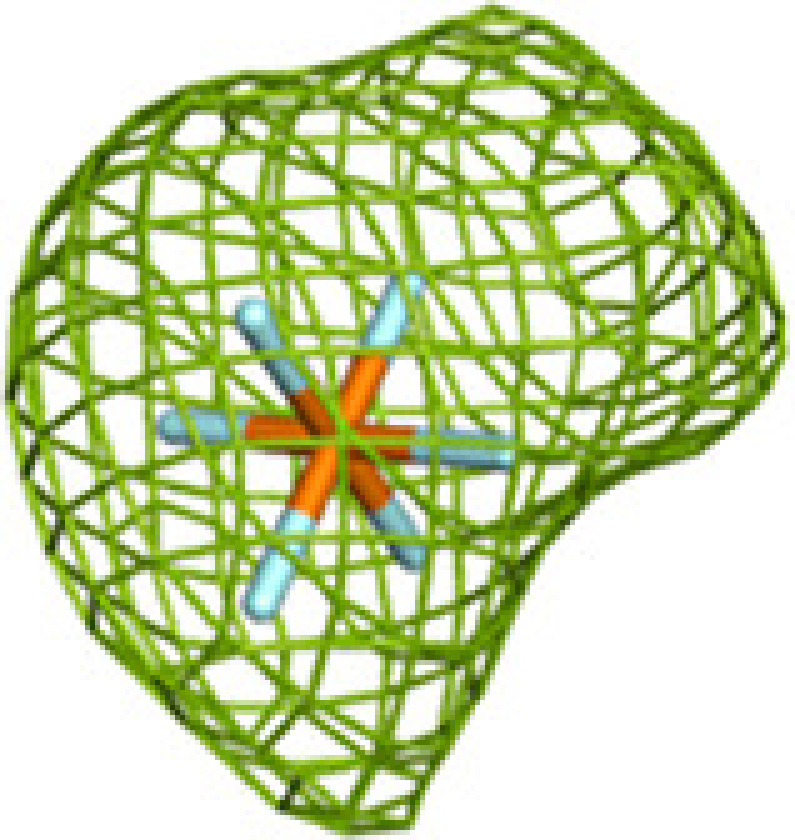
	H1	M-83 (B811, A/B) (double conformation)	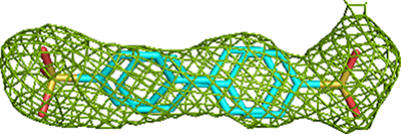
	H2	M-83 (A813)	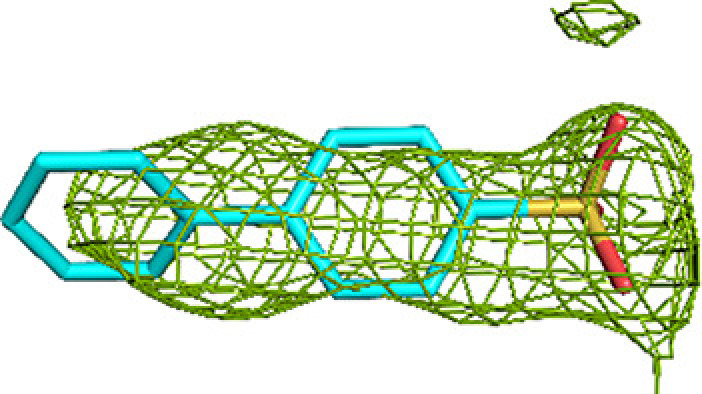
	H3	M-83 (A809)	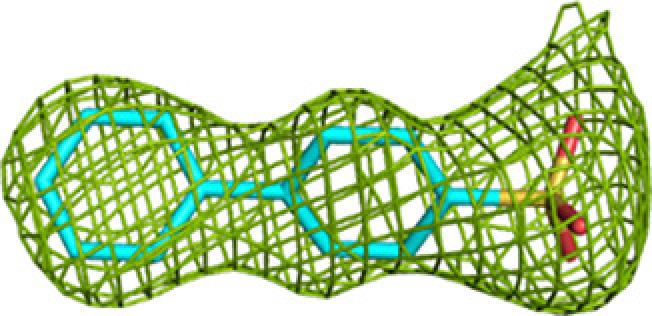
KAT	K1	No binding	—
	K2/K3	M-83 (C508)	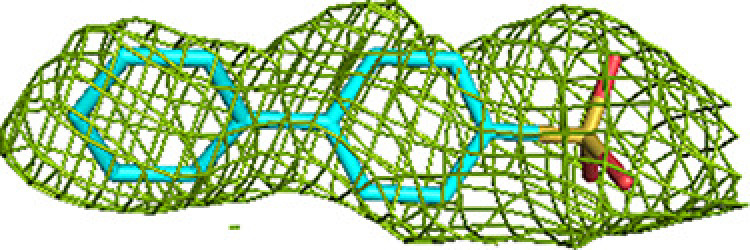

**Table 3:** Representative omit *mF*_o_ − *DF*_c_ difference maps of fragment-binding events as observed at the CoA-C(ECH/HAD) site at the subsites defined in Table 4 ▸ The contour level of these maps is at 2.5σ. Further information on the listed fragments is provided in Supplementary Table S1.

Binding site	Subsite	Residue	Omit *mF*_o_ − *DF*_c_ difference map
CoA-C(ECH/HAD)	I2 (center)	B-E1 (B807)	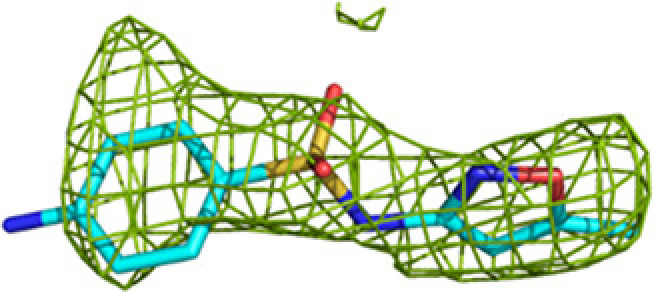
	I1 (to ECH)	B-H11 (B806)	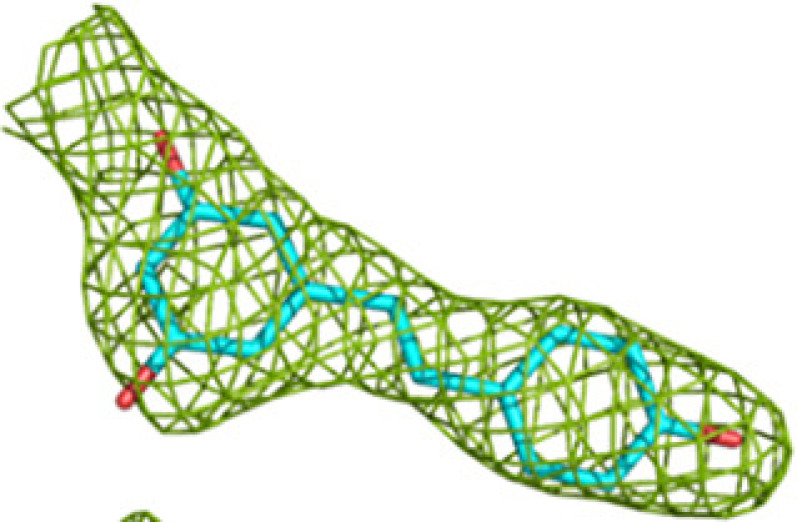
	H1 (to HAD)	M-76 (A810)	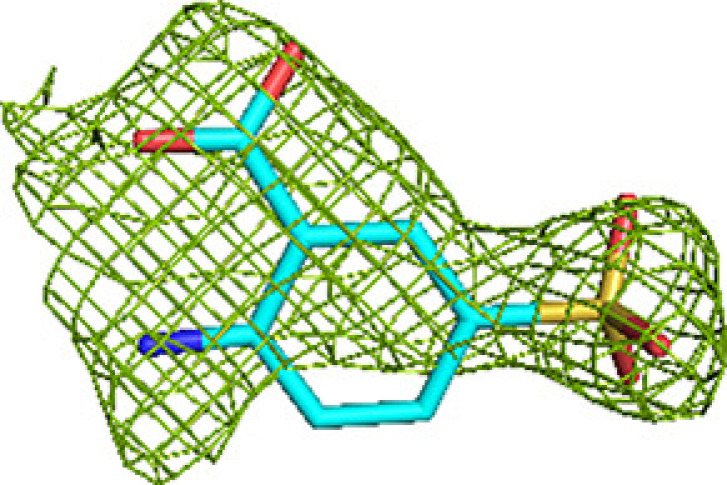
	I3 (to KAT)	M-72 (A810)	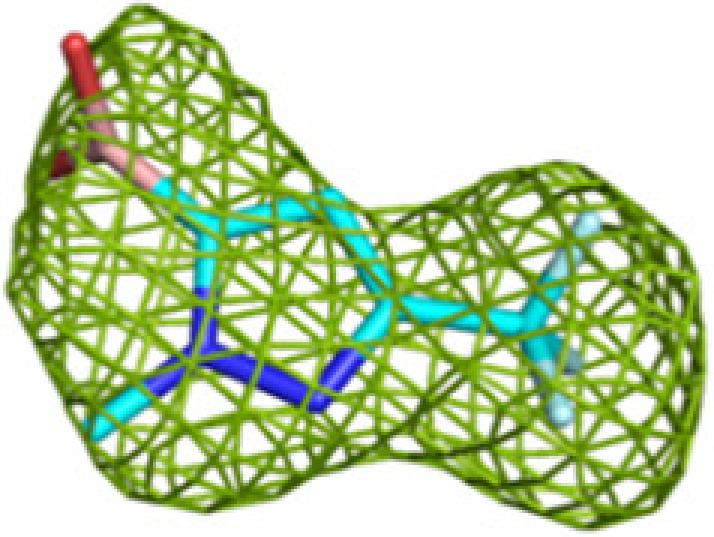

**Table 4:** Description of the 15 subsites as deduced from previous binding studies (Dalwani *et al.*, 2021 ▸)

Site	Subsite	Description of subsite
ECH active site	E1	Pantetheine binding pocket
	E2	Catalytic site
	E3	Acyl-tail binding tunnel
HAD active site	H0	NAD binding pocket
	H1	Pantetheine binding site, extending from the I2 site of the CoA-C(ECH/HAD) site
	H2	Catalytic site
	H3	Acyl-tail binding groove
KAT active site	K1	Pantetheine binding pocket
	K2	Catalytic site
	K3	Acyl-tail binding tunnel
CoA-C(ECH/HAD) site	I1	Subsite extending from I2 towards the ECH active site, branching off from the central region of I2
	I2	The central region of the CoA-C(ECH/HAD) binding site
	I3	Subsite extending from I2 towards the KAT active site, extending beyond the region that binds the S atom of the CoA-C molecule
CoA-A(HAD/KAT) site	C1	CoA binding site between the α and β subunits at the interface of the α and β subunits
CoA-B(ECH2) site	C2	CoA binding site on the α subunit on the opposite side to the ECH active site

**Table 5:** Summary of the fragment-binding events at the 15 subsites The numbers represent the number of binding events associated with the respective binding subsite. The subsites are described in Table 4 ▸. The binding mode of some fragments overlaps with two subsites; in such cases only one subsite is mentioned. In most cases binding events occur in pairs, as they occur in both copies of the two αβ dimers that form the asymmetric unit. Detailed information about the fragments is provided in Supplementary Table S1. Only two binding events at the subsites (out of 88) occur at a crystal contact, which are the two binding events of M-80 at the H0 subsite (Supplementary Table S4). Four binding events at the subsites (out of 88) involve double conformations (M-53 and M-83; Supplementary Table S4).

	ECH active site			HAD active site				KAT active site			CoA-C†			CoA-A†	CoA-B†		
Subsites	E1	E2	E3	H0	H1	H2	H3	K1	K2	K3	I1	I2	I3	C1	C2	Other	Total
B-E1						2				2		1					5
B-H11		2									2						4
B-51														2			2
B-B3																2	2
B-77																1	1
M-1		2	2	2		2				2		6				9	25
M-10	2															1	3
M-49	2											2				3	7
M-53		2					2					2	2		1	2	11
M-72	2		4								1	2	2				11
M-76	2				1											11	14
M-79	2															2	4
M-80		2	2	2													6
M-83		2	2		2	2	1			2						2	13
M-92		2	4														6
M-109	2		3				2										7
Totals	12	12	17	4	3	6	5			6	3	13	4	2	1	33	121
Totals	41			18				6			20			2	1	33	121

† CoA-A, CoA-B and CoA-C refer to the CoA-A(HAD/KAT), CoA-B(ECH2) and CoA-C(ECH/HAD) sites.
