# Supplementary material for: Crystallographic fragment-binding studies of the Mycobacterium tuberculosis trifunctional enzyme suggest binding pockets for the tails of the acyl-CoA substrates at its active sites and a potential substrate-channeling path between them
Source: Acta Crystallogr D Struct Biol. 2024 Jul 16;80(Pt 8):605–19. doi: 10.1107/S2059798324006557 (PMC11301753; doi:10.1107/S2059798324006557)
Supplement: Supplementary file 1 [file d-80-00605-sup1.pdf]

STRUCTURAL
BIOLOGY

Volume 80 (2024)

Supporting information for article:

Crystallographic fragment-binding studies of the *Mycobacterium tuberculosis* trifunctional enzyme suggest binding pockets for the tails of the acyl-CoA substrates at its active sites and a potential substrate-channeling path between them

Subhadra Dalwani, Alexander Metz, Franziska U. Huschmann, Manfred S. Weiss, Rik K. Wierenga and Rajaram Venkatesan

Table S1 Summary of the molecular properties of the 16 fragments for which binding has been observed

The 5 HZB compounds (B-XX) are hits from the first screen and the 11 Marburg compounds (M-XX) have been obtained from the second screen. Representative omit mFo-DFc difference maps of binding events of each of the fragments are shown in the last column of this table. The contour level of these difference maps is 2.5sigma.

Identifier	Resolution of structure	Mw(Da)	Predicted overall charge of predominant form at pH 7	Structural formula	Number of molecules bound	Stock solution	Omit mF_o-DF_c difference map (residue number)
B-E1 (HZB) (8OPU)	3.05	253.28	0	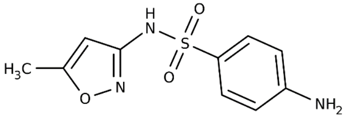	5	1 M in 100% DMSO (pre-spotted)[†]	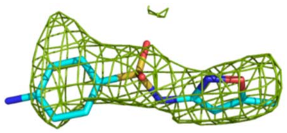 (B807)
B-H11 resveratrol (HZB) (8OPV)	2.8	194.19	0	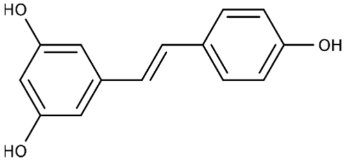	4	1 M in 100% DMSO (pre-spotted)[†]	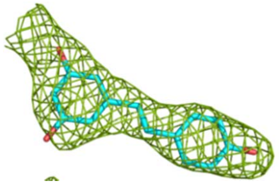 (B806)

B-51 caffeine (HZB) (8OPW)	2.52	228.24	0	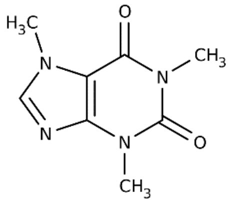	2	1 M in 100% DMSO (pre-spotted)†	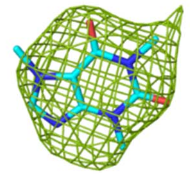 (B801)
B-B3 trehalose (HZB) (8OPX)	2.9	252.14	0	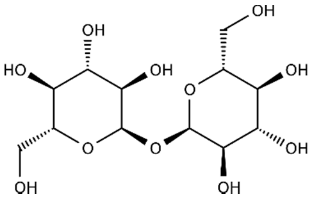	2	1 M in 100% DMSO (pre-spotted)†	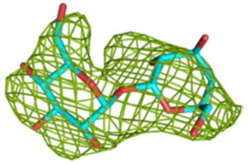 (G1, G2)
B-77 (HZB) (8OPY)	2.45	342.3	0	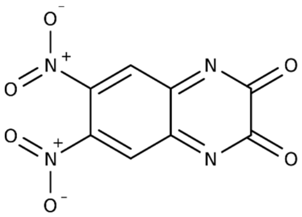	1	1 M in 100% DMSO (pre-spotted)†	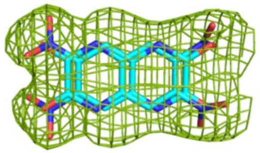 (D501, double conformation)
M-1 (Marburg) (8OQL)	2.7	144.96	-1	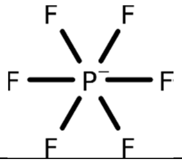	25	0.5 M in 100% DMSO	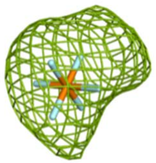

							(A814)
M-10 (Marburg) (8OQM)	3.2	239.24	-1	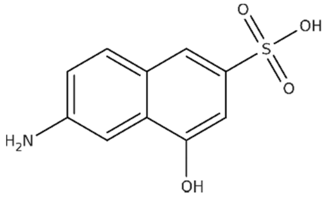	3	0.5 M in 50% DMSO	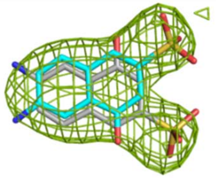 (D505, with symmetry mate in grey)
M-49 (Marburg) (8OQO)	2.6	159.17	-1	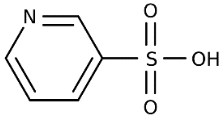	7	0.5 M in 50% DMSO	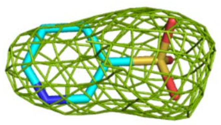 (C505)
M-53 (Marburg) (8OQN)	2.2	202.21	-1	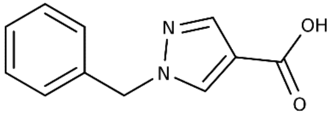	11	0.5 M in 100% DMSO	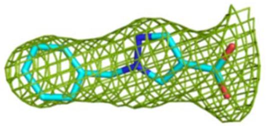 (B811)
M-72 (Marburg) (8PF8)	2.24	193.92	-1	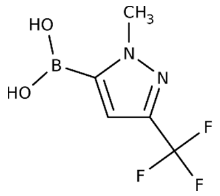	11	0.5 M in 100% DMSO	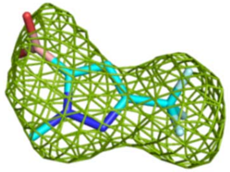 (A810)

M-76 (Marburg) (8OQP)	2.19	217.2	-2	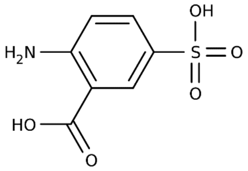	14	0.5 M in 100% DMSO	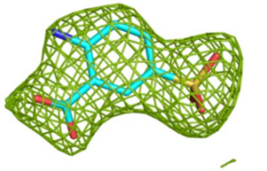 (A808)
M-79 (Marburg) (8OQQ)	2.59	238.62	-2	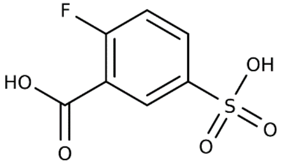	4	0.5 M in 100% DMSO	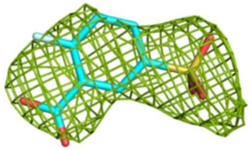 (A805)
M-80 (Marburg) (8OQR)	2.4	183.19	-1	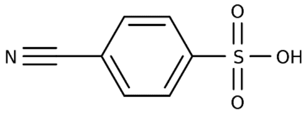	6	0.5 M in 100% DMSO‡	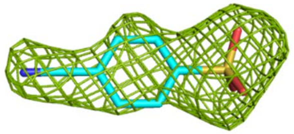 (A810)
M-83 (Marburg) (8OQS)	2.33	234.27	-1	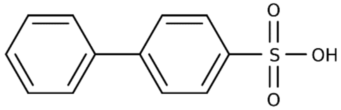	13	0.25 M in 50% DMSO‡	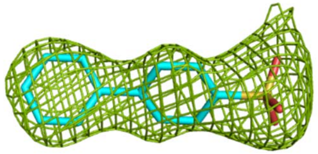 (A809)
M-92 (Marburg) (8OQU)	2.89	192.62	-1	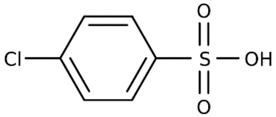	6	0.5 M in aqueous solution	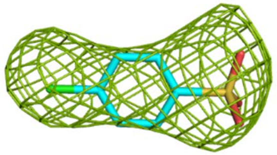

							(A810)
M-109 (Marburg) (8OQV)	2.78	203.17	-1	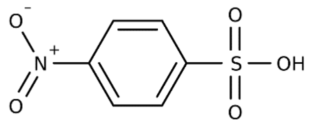	7	0.5 M in aqueous solution	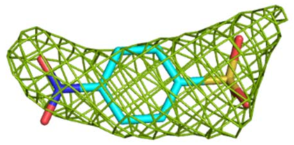<br>(B804)

† For these experiments pre-spotted plates were used, as provided by HZB.

‡ These compounds were provided as aqueous solution and subsequenly dried and redissolved in the DMSO.solvent.

Table S2 Data collection and refinement statistics for the HZB fragments.

Data set	B-E1	B-H11	B-51	B-B3	B-77
**Data collection**					
Beam line	BESSY II (14.1)	BESSY II (14.1)	BESSY II (14.1)	BESSY II (14.1)	BESSY II (14.1)
Detector	Pilatus 6M	Pilatus 6M	Pilatus 6M	Pilatus 6M	Pilatus 6M
Wavelength (Å)	0.8950	0.8950	0.8950	0.8950	0.8950
Temperature (K)	100	100	100	100	100
**Data Processing**					
Space group	C2	C2	C2	C2	C2
Unit cell parameters					
a,b,c (Å)	248.76, 135.08, 119.74	249.69, 134.68, 119.48	249.51, 134.50, 118.98	248.41, 135.16, 119.16	249.83, 134.53, 118.84
α,β,γ (°)	90.0, 110.61, 90.0	90.0, 110.56, 90.0	90.0, 110.86, 90.0	90.0, 110.56, 90.0	90.0, 110.82, 90.0
Processing software	xdsapp [1], [2]	xdsapp [1], [2]	xdsapp [1], [2]	xdsapp [1], [2]	xdsapp [1], [2]
Resolution range (Å)[&]	48.42-3.04 (3.23-3.04)	48.38-2.8 (2.97-2.80)	48.19-2.51 (2.67-2.51)	48.37-2.9 (3.07-2.90)	48.21-2.45 (2.59-2.45)
R_{pim} (%) (all I+/I-)[&]	14.4 (77.9)	10.5 (87.6)	12.7 (172.6)	7.9 (60.2)	8.9 (171.1)
$CC_{1/2}$ (%)[&]	98.4 (53.8)	98.8 (50.4)	99.0 (14.4)	99.4(63.9)	99.5 (20.0)
$\langle I/\sigma(I)\rangle$ [&]	6.48 (1.32)	6.88 (1.14)	6.00 (0.49)	10.24 (1.54)	7.42 (0.53)
Completeness (%)[&]	99.4 (98.1)	99.1 (98.2)	97.5 (89.6)	97.7 (97.1)	98.2 (94.4)
Multiplicity[&]	6.4 (6.4)	4.8 (5.0)	3.8 (3.4)	3.9 (3.9)	3.8 (3.5)
No. of reflections	450495	439437	462833	309390	505899
No. of unique reflections	70733	90705	121668	80269	133151
Wilson *B* factor (Å^2)[#]	65.4	61.5	57.2	59.4	57.6
**Refinement Statistics**					
Resolution range (Å)	46.59 - 3.05	48.38 - 2.8	48.19 - 2.52	48.37 - 2.9	47.0 - 2.45
No. of used reflections	70654	90660	120659	80266	132305
R_{work} (%)	20.79	20.34	20.46	18.85	21.08
R_{free} (%)	24.61	23.03	23.97	22.37	24.35
Total No. of atoms	17000	16953	17040	16841	17025
No. of waters	66	132	174	46	132
Average B factors (Å^2)					
Protein	69.78	68.53	66.42	68.85	66.13
Others	94.83	94.82	100.81	105.39	100.19
Waters	50.41	52.83	52.23	45.74	54.31
R.m.s. deviations					
Bond lengths (Å)	0.0016	0.0017	0.0021	0.0023	0.0025
Bond angles (°)	0.42	0.46	0.47	0.53	0.51
Ramachandran plot (%)[3]					
Favored	96.02	96.31	96.77	96.16	96.78

Allowed	4.02	3.42	3.01	3.83	3.00
Outliers	0.27	0.27	0.22	0.05	0.22
**PDB ID**	8OPU	8OPV	8OPW	8OPX	8OPY

& The highest resolution shell is given in parentheses.

As reported in the PDB validation report.

Table S3 Data collection and refinement statistics for the Marburg fragments.

Data set	M-1	M-10	M-49	M-53	M-72	M-76
**Data collection**						
Beam line	MAX IV (BioMAX)	MAX IV (BioMAX)	Petra (P13)	Petra (P13)	DLS (I04-1)	DLS (I03)
Detector	Eiger 16M Hybrid-pixel	Eiger 16M Hybrid-pixel	PILATUS 6M	PILATUS 6M	Pilatus 6M-F	Eiger2 XE 16M
Wavelength (Å)	0.968619	0.968619	0.9198	0.9198	0.91589	0.976254
Temperature (K)	100	100	100	100	100	100
**Data Processing**						
Space group	C2	C2	C2	C2	C2	C2
Unit cell parameters						
a,b,c (Å)	249.46, 134.25, 118.4	247.92, 134.91, 119.21	250.78, 136.14, 121.05	247.31, 134.03, 116.41	250.96, 134.61, 119.97	250.93, 132.8, 119.29
α,β,γ (°)	90.0, 110.74, 90.0	90.0, 110.62, 90.00	90.0, 110.42, 90.0	90.0, 109.69, 90.0	90.0, 110.44, 90.0	90.0, 110.46, 90.0
Processing software	xds [4], AIMLESS [5]	xds [4], AIMLESS [5]	xds [4], AIMLESS [5]	xds [4], AIMLESS [5]	autoPROC[6], STARANISO[7]	autoPROC[6], STARANISO[7]
Resolution range (Å) &	48.32-2.7 (2.75-2.7)	48.29-3.20 (3.28-3.2)	117.8-2.6 (2.64-2.6)	116.42-2.2 (2.24-2.2)	112.42-2.23 (2.47-2.24)	117.55-2.19 (2.35-2.19)
R_{pim} (%) (all I+/I-)&	4.6 (59.1)	12 (125.3)	4.8 (65.6)	4.7 (85.1)	4.4 (46.3)	4.1 (53.5)
$CC_{1/2}$ (%)&	99.8 (53.8)	98.6 (24.1)	99.6 (45.3)	99.8 (34.5)	99.8 (62.3)	99.9 (55)
<I/σ(I)> &	10.9 (1.3)	5.9 (1.1)	9.3 (1.0)	9 (1.1)	11.5 (1.6)	13.3 (1.5)
Completeness (%)&	99.7 (95.9)	99.5 (97)	99.6 (92.1)	99.7 (94.9)	95.7 (74)	95.3 (65.3)
Multiplicity&	4.8 (4.8)	3.8 (3.8)	6.7 (6.2)	6.8 (6.6)	5.3 (5.6)	7.0 (7.0)
No. of reflections	486278	228561	784947	1223214	701657	1017298
No. of unique reflections	100954	60500	116826	179810	131177	145082
Wilson *B* factor ($Å^2$)#	71.6	94.9	71.1	52.4	46.8	46.5
**Refinement Statistics**					46.8	
Resolution range (Å)	48.32 - 2.7	48.29 - 3.2	58.75 - 2.6	48.98 - 2.2	112.42 - 2.24	27.08 - 2.19
No. of used reflections	100937	60087	116686	179611	131167	145050
R_{work} (%)	18.37	20.34	18.81	19.27	18.93	19.60
R_{free} (%)	21.88	24.19	22.48	22.12	21.88	22.41
Total No. of atoms	17100	16898	17006	17291	17613	17630

No. of waters	64	50	182	188	491	575
Average B factors (Å^2)						
Protein	73.35	100.74	85.28	69.73	58.07	53.99
Others	113.29	126.47	117.74	96.58	81.94	77.95
Waters	59.01	68.38	66.32	51.83	48.15	49.21
R.m.s. deviations						
Bond lengths (Å)	0.0019	0.0016	0.0017	0.0021	0.0021	0.0025
Bond angles (^{O})	0.47	0.44	0.45	0.53	0.52	0.54
Ramachandran plot (%)						
Favored	96.85	96.59	96.40	96.56	96.93	96.79
Allowed	3.01	3.28	3.24	3.25	2.85	3.03
Outliers	0.13	0.13	0.36	0.18	0.22	0.18
**PDB ID**	8OQL	8OQM	8OQO	8OQN	8PF8	8OQP

[&] The highest resolution shell is given in parentheses.

[#] As reported in the PDB validation report.

Data set	M-79	M-80	M-83	M-92	M-109
**Data collection**					
Beam line	MAX IV (BioMAX)	MAX IV (BioMAX)	MAX IV (BioMAX)	MAX IV (BioMAX)	MAX IV (BioMAX)
Detector	Eiger 16M Hybrid-pixel	Eiger 16M Hybrid-pixel	Eiger 16M Hybrid-pixel	Eiger 16M Hybrid-pixel	Eiger 16M Hybrid-pixel
Wavelength (Å)	0.976254	0.976254	0.976254	0.976254	0.976254
Temperature (K)	100	100	100	100	100
**Data Processing**					
Space group	C2	C2	C2	C2	C2
Unit cell parameters					
a,b,c (Å)	250.34, 134.02, 119.75	250.70, 135.61, 120.89	250.46, 134.70, 119.30	249.46, 134.89, 119.48	248.79, 135.63, 119.69
α,β,γ (^{O})	90.0, 110.53, 90.0	90.0, 110.27, 90.0	90.0, 110.68, 90.0	90.0, 110.47, 90.0	90.0, 110.63, 90.0
Processing software	autoPROC[6], STARANISO[7]	autoPROC[6], STARANISO[7]	autoPROC[6], STARANISO[7]	autoPROC[6], STARANISO[7]	EDNAproc[8], STARANISO[7]
Resolution range (Å) [&]	117.2-2.59 (2.82-2.59)	117.59-2.4 (2.64-2.4)	117.16-2.33 (2.49-2.33)	116.86-2.89 (3.17-2.89)	45.51-2.77 (2.9-2.77)
R_{pim} (%) (all I+/I-)[&]	11.9 (143.2)	11.8 (126.6)	6.1 (49.3)	7.9 (57.1)	7.8 (74.9)
$CC_{1/2}$[&]	98 (25.1)	98.2 (33.9)	99.4 (59.1)	99.2 (49.8)	99.3 (41.2)
$\langle I/\sigma(I)\rangle$ [&]	7.2 (1.5)	5.9 (1.5)	9.1 (1.5)	8.5 (1.5)	8.3 (1.1)
Completeness (%)[&]	92.2 (53.7)	91.8 (52.1)	92.9 (60.9)	93.2 (65.7)	91.9 (52.3)
Multiplicity[&]	6.7 (7.2)	6.5 (7.2)	7.1 (6.2)	6.9 (7.3)	6.7 (6.8)
No. of reflections	601604	773421	801947	446439	540036
No. of unique reflections	89654	118277	113738	64333	80655

Wilson *B* factor (Å^2)#	55.1	48	40.2	77.7	70.5
**Refinement Statistics**					
Resolution range (Å)	50.27 - 2.59	47.92 - 2.4	55.81 - 2.33	116.86 - 2.89	45.51 - 2.78
No. of used reflections	89609	118191	113689	64297	80595
R_{work} (%)	19.63	21.97	17.77	17.99	19.74
R_{free} (%)	23.12	25.96	21.80	21.72	23.17
Total No. of atoms	17006	17260	17963	16964	16903
No. of waters	186	444	681	61	47
Average B factors (Å^2)					
Protein	65.82	54.80	50.66	76.08	72.08
Others	96.59	75.52	79.45	104.32	108.66
Waters	49.08	79.66	46.30	52.51	49.04
R.m.s. deviations					
Bond lengths (Å)	0.0020	0.0024	0.0033	0.0019	0.0019
Bond angles (^{O})	0.49	0.51	0.58	0.47	0.48
Ramachandran plot (%)					
Favored	95.87	96.54	96.82	95.66	95.78
Allowed	3.95	3.24	3.05	4.16	4.02
Outliers	0.18	0.22	0.13	0.18	0.22
**PDB ID**	8OQQ	8OQR	8OQS	8OQU	8OQV

[&] The highest resolution shell is given in parentheses.

[#] As reported in the PDB validation report.

Table S4 Summary of the fragment binding events at the subsites defined in Table 4.

Fragment	Crystal contact	occupancy	RSCC[#]	ECH active site			HAD active site				KAT active site			CoA-A[*]	CoA-B[*]	CoA-C[*]			other
				E1	E2	E3	H0	H1	H2	H3	K1	K2	K3	C1	C2	I1	I2	I3	
B-E1																			
O8D-B807		0.78	0,8														yes, B		
O8D-B808		0.78	0,91						yes, B										
O8D-D508		0.83	0,94										yes, D						
O8D-C507		0.83	0,94										yes, C						
O8D-A806		0.78	0,86						yes, A										
B-H11																			
STL-A805		1.00	0,89		yes, A														
STL-A806		1.00	0,9													yes, A			
STL-B805		1.00	0,86		yes, B														
STL-B806		1.00	0,92													yes, B			
B-51																			
CFF-D501		1.00	0,93											yes, D					
CFF-B801		1.00	0.93											yes, C					
B-B3																			
GLC-(H1, H2)		0.6/0.6	0,86/0,86																yes, D
GLC-(G1, G2)		0.8/0.8	0,85/0,83																yes, C
B-77																			
DNQ(D501,D502)	yes	0.25/0.25	0,81/0,79																yes, D
M-1																			
A9J-A807		1.00	0,97			yes, A													
A9J-A808		1.00	0,96																yes, A
A9J-A809		1.00	0,94														yes, A		
A9J-A810		1.00	0,97																yes, A
A9J-A811		1.00	0,97		yes, A														
A9J-A812		1.00	0,94														yes, A		
A9J-A813		1.00	0,96						yes, A										
A9J-A814		1.00	0,94				yes, A												
A9J-A815		1.00	0,95																yes, A
A9J-A816		1.00	0,9														yes, A		
A9J-B808		1.00	0,96			yes, B													
A9J-B809		1.00	0,99																yes, B
A9J-B810		1.00	0,98				yes, B												

A9J-B811		1.00	0,93									yes, B
A9J-B812		1.00	0,93							yes, B		
A9J-B813		1.00	0,98		yes, B							
A9J-B814		1.00	0,94							yes, B		
A9J-B815		1.00	0,9							yes, B		
A9J-B816		1.00	0,96			yes, B						
A9J-C509		1.00	0,99									yes, C
A9J-D508		1.00	0,98					yes, D				
A9J-C510		1.00	0,98					yes, C				
A9J-D509		1.00	0,99									yes, D
A9J-D510	yes	1.00	0,9									yes, D
A9J-B817		1.00	0,82									yes, B
M-10												
A1IDU-A805		1.00	0,87	yes,A								
A1IDU-B804		1.00	0,85	yes, B								
VWO-D505	yes	0.5	0,94									yes, D
M-49												
VXH-A2405		1.00	0,93	yes,A								
VXH-A2406		1.00	0,89							yes, A		
VXH-B2405		1.00	0,95	yes, B								
VXH-B2406	yes	1.00	0,93									yes, B
VXH-B2407		1.00	0,88							yes, B		
VXH-C505		1.00	0,92									yes, C
VXH-D506(A,B)		0.5/0.5	0.88/0.88									yes, D
M-53												
W3U-A805		1.00	0,91									yes, A
W3U-A806		1.00	0,95		yes, A							
W3U-A807(A,B)		0.5/0.5	0,84/0,84								yes, A	
W3U-A808		1.00	0,75				yes, A					
W3U-B808		1.00	0,87							yes, B		
W3U-B809(A,B)		0.5/0.5	0,83/0,83								yes, B	
W3U-B810		1.0	0,85									yes, B
W3U-B811		1.00	0,94		yes, B							
W3U-B812		1.00	0,89						yes, B			
W3U-B813		1.00	0,86				yes, B					
W3U-A809		1.00	0,72							yes, A		
M-72												
JXL-B809		1.00	0,9								yes, B	
JXL-B808		1.00	0,86	yes, B								

JXL-B810		1.00	0,94			yes, B							
JXL-A810		1.00	0,92									yes, A	
JXL-A809		1.00	0,81	yes,A									
JXL-A811		1.00	0,95			yes, A							
YLN-A808		1.00	0,96			yes, A							
YMK-A806		1.00	0,92							yes, A			
YLZ-A807		1.00	0,95								yes, A		
YLN-B806		1.00	0,96			yes, B							
YLZ-B805		1.00	0,92								yes, B		
M-76													
VXZ-A807		1.00	0,95	yes,A									
VXZ-A808	yes	1.00	0,98										yes, A
VXZ-A809	yes	1.00	0,95										yes, A
VXZ-A810		1.00	0,79					yes, A					
VXZ-B806	yes	1.00	0,98										yes, B
VXZ-B807		1.00	0,96	yes, B									
VXZ-B808	yes	1.00	0,88										yes, B
VXZ-C505		1.00	0,94										yes, C
VXZ-C506		1.00	0,9										yes, C
VXZ-D506		1.00	0,95										yes, D
VXZ-D507		1.00	0,95										yes, D
VXZ-D508		1.00	0,88										yes, D
VXZ-B809		1.00	0,94										yes, B
VXZ-A811		1.00	0,95										yes, A
M-79													
VWE-A805		1.00	0,95	yes,A									
VWE-A806	yes	1.00	0,94										yes, A
VWE-B808		1.00	0,91	yes, B									
VWE-B809	yes	1.00	0,95										yes, B
M-80													
VWT-A808		1.00	0,89			yes, A							
VWT-A809		1.00	0,9		yes, A								
VWT-B807		1.00	0,83			yes, B							
VWT-B808		1.00	0,94		yes, B								
VWT-A810	yes	1.00	0,95				yes, B						
VWT-A811	yes	1.00	0,98				yes, A						
M-83													
VWZ-A809		1.00	0,95						yes, A				
VWZ-A810(A,B)		0.5/0.5	0,85/0,85					yes, A					

VWZ-A811		1.00	0,87			yes, A													
VWZ-A812		1.00	0,91		yes, A														
VWZ-A813		1.00	0,81						yes, A										
VWZ-B811(A,B)		0.5/0.5	0,91/0,91					yes, B											
VWZ-B812		1.00	0,86			yes, B													
VWZ-B813		1.00	0,89		yes, B														
VWZ-B814		1.00	0,82						yes, B										
VWZ-C508		1.00	0,9										yes, C						
VWZ-D507		1.00	0,89										yes, D						
VWZ-B815	yes	1.00	0,9																yes, B
VWZ-A814	yes	1.00	0,92																yes, A
M-92																			
VXN-B807		1.00	0,93			yes, B													
VXN-B808		1.00	0,89			yes, B													
VXN-A809		1.00	0,96			yes, A													
VXN-B809		1.00	0,82		yes, B														
VXN-A810		1.00	0,92			yes, A													
VXN-A811		1.00	0,85		yes, A														
M-109																			
VWI-A806		1.00	0,89							yes, A									
VWI-A807		1.00	0,91			yes, A													
VWI-B804		1.00	0,93			yes, B													
VWI-A808		1.00	0,76	yes,A															
VWI-B805		1.00	0,79	yes, B															
VWI-A809		1.00	0,85			yes, A													
VWI-B806		1.00	0,86							yes, B									
				E1	E2	E3	H0	H1	H2	H3	K1	K2	K3	C1	C2	I1	I2	I3	other
Totals	14		121	12	12	17	4	3	6	5		0	6	2	1	3	13	4	33
					ECH				HAD			KAT		CoA-A*	CoA-B*		CoA-C*		
Totals					41				18			6		2	1		20		

[#] As reported in the PDB validation report.

* CoA-A, CoA-B, CoA-C refer to the CoA-A(HAD/KAT), CoA-B(ECH2) and CoA-C(ECH/HAD) regions, respectively.

Table S5 Analysis of the CoA-protein interactions of CoA bound in the active sites and in the additional binding sites.†

**Active sites**	**PDB ID/ residue number**	**SC/ total**	**SC perentage**	**S-S interactions**	**Salt bridge interactions**	**H-bond/ total**	**H-bond percentage**	**Interactions of the adenine moiety**
CoA(ECH) - chain A (bent conformation)	7O4T/A801	15/23		0	0	9/23		Stacking of adenine between side chains of Pheα304 and Valα29
CoA(ECH) - chain B (bent conformation)	7O4T/B801	20/30		0	0	12/30		
CoA(KAT) - chain C (extended conformation)	7O4T/C501	19/24		1	0	6/24		Stacking of adenine between side chains of Argβ210 and Leuβ221
CoA(KAT) - chain D (extended conformation)	7O4T/D501	26/33		1	1 (weak)	7/33		
CoA active sites, totals:								
CoA (total)		80/110	73%			34/110	31%	
CoA (average)		20.0/27.5	73%			8.5/27.5	31%	
**Additional binding sites**								
CoA-A(HAD/KAT) chains BC	7O4R/D506	30/31		0	1	4/31		Stacking of adenine between side chains of Trpβ244 and Glnα629
CoA-A(HAD/KAT) chains AD	7O4R/C508	25/25		0	1	3/25		
CoA-B(ECH2) chain A	7O4S(A809	23/27		0	1	5/27		Stacking of adenine between pantetheine moiety and bulk solvent
CoA-B(ECH2) chain B	7O4S/B808	18/25		0	1	6/25		
CoA-C(ECH/HAD) chain A	7O4T/A802	14/24		0	0	1/24		Stacking of adenine between side chain of Lysα469 and bulk solvent

CoA-C(ECH/HAD) chain B	7O4T/B803	5/8		0	0	0/8	
CoA additional binding sites, totals:							
CoA (total)		115/140	82%			19/140	14%
CoA (average)		19.2/23.3	82%			3.2/23.3	14%

†The contact distance cut-off value has been 3.7Å. None of the additional CoA binding sites are near crystal contacts. SC refers to interactions with side chains. H-bond refers to hydrogen bond interactions. S-S interactions refer to interactions between two sulfur atoms. These S-S interactions are only observed in the thiolase active site and they have been classified as Van der Waals interactions (in both active sites of the thiolase dimer, it concerns interactions with the sulfur atom of the nucleophilic cysteine (at 3.2Å); the other, nearest sulfur is from Cys389, the acid/base cysteine, which is at 5Å). Hydrogen bond interactions are counted after visual inspection.

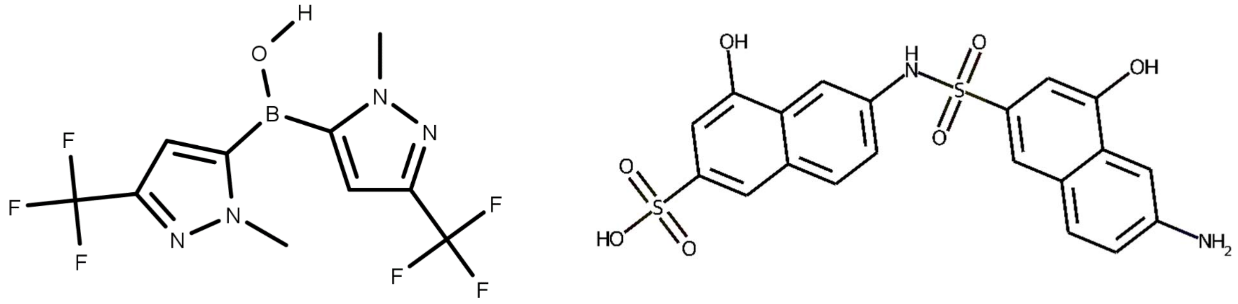

Figure S1 The covalent structure of the M-72 dimer (left panel) and the covalent structure of the M-10 dimer (right panel).

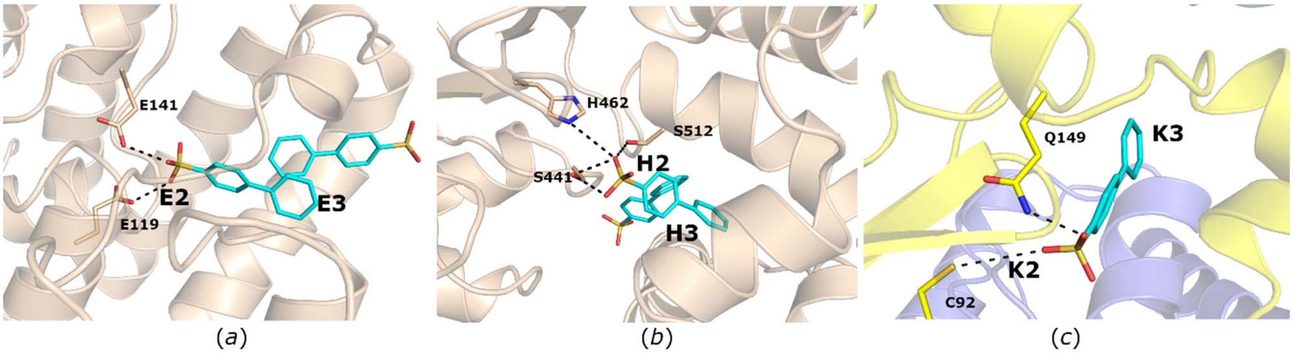

Figure S2 The mode of binding of fragment M-83 at the ECH, HAD and KAT catalytic sites is shown. Dotted lines visualize the described interactions. (a) In the ECH catalytic site (E2) the sulfonate group of M-83 interacts with the side chains of the catalytic glutamates, Gluα119 and Gluα141. The second binding event of M-83 also visualises the acyl-tail binding tunnel, E3 (Fig. 4A). (b) In the HAD catalytic site (H2) the sulfonate group of M-83 interacts with the side chains of Hisα462, Serα441 and Serα512. The second binding event of M-83 visualises also the acyl-tail binding tunnel, H3 (Fig. 4B) (c) In the KAT catalytic site (K2) the sulfonate group of M-83 interacts with the side chain of Glnβ149 and there is also a weak interaction with the side chain of the nucleophilic cysteine, Cysβ92, whereas its aromatic part visualizes the acyl-tail binding tunnel (K3).

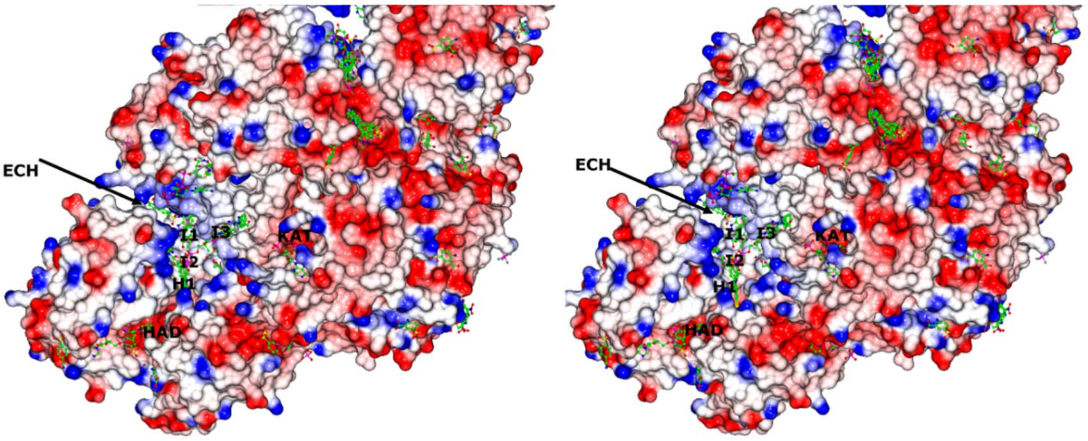

Figure S3 Stereo image of the protein surface of the possible substrate channeling path between the three active sites of MtTFE, as also shown in Fig. 5. The image shows the electrostatic properties of the molecular surface of the CoA-C(ECH/HAD) region, near the interface of chain A (α subunit) and chain D (β subunit) of the CoA-C structure (PDB ID 7O4T), color coded such that the blue and red colors identify surface regions with positive and negative electrostatic potential, respectively. Neutral regions have a white color. The ligands included are also shown in Movie S1. The locations of the catalytic sites are labeled as ECH (with arrow), HAD and KAT. The CoA-C(ECH/HAD) site is labeled as I2, whereas the regions extending to the ECH, HAD and KAT catalytic sites are labeled as I1, H1, and I3, respectively.

Movie S1. The fragment binding events at the surface region near the CoA-C(ECH//HAD) binding site between the ECH, HAD and KAT active sites. The electrostatic surface is calculated from the CoA-C structure (PDP ID 7O4T), color coded such that the blue and red colors identify surface regions with positive and negative electrostatic potential, respectively. In the very first frames the MtTFE cartoon structure is shown, together with ligands bound in the ECH active site (CoA), HAD active site (NAD^+) and KAT active site (CoA) as well as the CoA bound in the three additional CoA binding sites. All 121 fragment binding events are included in the subsequent frames. The view is similar as in Fig. 1 and Fig. 5.

References

[1] Krug, M., Weiss, M. S., Heinemann, U. & Mueller, U. (2012). *J. Appl. Crys.* **45**, 568–572.
[2] Sparta, K. M., Krug, M., Heinemann, U., Mueller, U. & Weiss, M. S. (2016). *J. Appl. Cryst.* **49,** 1085–1092.
[3] Chen, V.B., Arendall, W. B. Headd, J. J., Keedy, D. A, Immormino, R.M,, Kapral, G.J., Murray, L.W., Richardson, J. S. & Richardson, D. C. (2010) *Acta Cryst.* D**66**, 12-21.
[4] Kabsch, W. (2010). *Acta Cryst.* D**66**, 125–132.

[5] Evans P. R. & Murshudov, G. N. *Acta Cryst.* D**69**, 1204–1214.
[6] Vonrhein, C., Flensburg, C., Keller, P., Sharff, A., Smart, O., Paciorek, W., Womack, T. & Bricogne, G. (2011). *Acta Cryst.* D**67**, 293–302.
[7 Tickle, I. J., Flensburg, C., Keller, P., Paciorek, W. Sharff, A., Vonrhein, C. & Bricogne, G. "STARANISO", Cambridge, United Kingdom. Global Phasing Ltd. (https://staraniso.globalphasing.org/cgi-bin/staraniso.cgi)
[8] Incardona, M. F., Bourenkov, G. P., Levik, K., Pieritz, R. A., Popov, A. N. & Svensson, O. (2009) *J. Synchrotron Rad.* **16**, 872-879.
